# Clinical impact of statin intensity according to age in patients with acute myocardial infarction

**DOI:** 10.1371/journal.pone.0269301

**Published:** 2022-06-15

**Authors:** Kyusup Lee, Myunhee Lee, Dae-Won Kim, Jinseob Kim, Sungmin Lim, Eun Ho Choo, Chan Joon Kim, Chul Soo Park, Hee Yeol Kim, Ki-Dong Yoo, Doo Soo Jeon, Kiyuk Chang, Ho Joong Youn, Wook-Sung Chung, Min Chul Kim, Myung Ho Jeong, Youngkeun Ahn, Jongbum Kwon, Mahn-Won Park

**Affiliations:** 1 Cardiovascular Research Institute for Intractable Disease, College of Medicine, The Catholic University of Korea, Seoul, Republic of Korea; 2 Department of Cardiology, Daejeon St. Mary’s Hospital, College of Medicine, The Catholic University of Korea, Daejeon, Republic of Korea; 3 Department of Epidemiology, School of Public Health, Seoul National University, Seoul, Republic of Korea; 4 Division of Cardiology, Department of Internal Medicine, Uijeongbu St Mary’s Hospital, The Catholic University of Korea, Uijeongbu, Republic of Korea; 5 Division of Cardiology, Department of Internal Medicine, Seoul St. Mary’s Hospital, The Catholic University of Korea, Seoul, Republic of Korea; 6 Division of Cardiology, Department of Internal Medicine, Yeouido St. Mary’s Hospital, The Catholic University of Korea, Seoul, Republic of Korea; 7 Division of Cardiology, Department of Internal Medicine, Bucheon St. Mary’s Hospital, The Catholic University of Korea, Bucheon, Republic of Korea; 8 Division of Cardiology, Department of Internal Medicine, St. Vincent’s hospital, The Catholic University of Korea, Suwon, Republic of Korea; 9 Division of Cardiology, Department of Internal Medicine, Incheon St. Mary’s Hospital, The Catholic University of Korea, Incheon, Republic of Korea; 10 Cardiovascular Center, Chonnam National University Hospital, Chonnam National University, Gwangju, Republic of Korea; 11 Department of Thoracic and Cardiovascular Surgery, Daejeon St. Mary’s Hospital, the Catholic University of Korea, Daejeon, Republic of Korea; Baylor Scott and White, Texas A&M College of Medicine, UNITED STATES

## Abstract

**Background:**

The available data are not sufficient to understand the clinical impact of statin intensity in elderly patients who undergo percutaneous coronary intervention (PCI) due to acute myocardial infarction (AMI).

**Methods:**

Using the COREA-AMI registry, we sought to compare the clinical impact of high- versus low-to-moderate-intensity statin in younger (<75 years old) and elderly (≥75 years old) patients. Of 10,719 patients, we included 8,096 patients treated with drug-eluting stents. All patients were classified into high-intensity versus low-to-moderate-intensity statin group according to statin type and dose at discharge. The primary end point was target-vessel failure (TVF), a composite of cardiovascular death, target-vessel MI, or target-lesion revascularization (TLR) from 1 month to 12 months after index PCI.

**Results:**

In younger patients, high-intensity statin showed the better clinical outcomes than low-to-moderate-intensity statin (TVF: 79 [5.4%] vs. 329 [6.8%], adjusted hazard ratio [aHR] 0.76; 95% confidence interval [CI] 0.59–0.99; P = 0.038). However, in elderly patients, the incidence rates of the adverse clinical outcomes were similar between two statin-intensity groups (TVF: 38 [11.4%] vs. 131 [10.6%], aHR 1.1; 95% CI 0.76–1.59; P = 0.63).

**Conclusions:**

In this AMI cohort underwent PCI, high-intensity statin showed the better 1-year clinical outcomes than low-to-moderate-intensity statin in younger patients. Meanwhile, the incidence rates of adverse clinical events between high- and low-to-moderate-intensity statin were not statistically different in elderly patients. Further randomized study with large elderly population is warranted.

## Background

For patients who experienced acute myocardial infarction (AMI), a pharmacologic therapy to prevent a recurrent cardiovascular events is a cornerstone of treatment [1]. Statin therapy has been shown to reduce major vascular events and vascular mortality in those individuals [2,3]. Thus, current guidelines recommend that high-intensity statin should be considered based on lowering low-density lipoprotein cholesterol (LDL-C) for secondary prevention in patients with atherosclerotic cardiovascular disease (ASCVD) [4–7].

Because of globally increasing number of adults aged 75 years or older and inherent their high cardiovascular risk and frailty [8–10], optimal treatment strategy is more required for elderly patients who underwent percutaneous coronary intervention (PCI) due to AMI. Recently, the European Society of Cardiology (ESC) and European Atherosclerosis Society (EAS) guideline recommends that, in older patients (>65 years old), maximal tolerated statin therapy be considered in the same way as for younger patients [11]. The American College of Cardiology (ACC)/American Heart Association (AHA) recommends that it is reasonable to continue high-intensity statin therapy after evaluation of the potential benefits versus adverse effect of statin therapy in patients older than 75 years of age with atherosclerotic cardiovascular disease (ASCVD) who are tolerating high-intensity statin therapy [12]. The National Lipid Association (NLA) recommends moderate- or high-intensity statin therapy for patients who are 75–80 years old and moderate-intensity statin therapy for patients who are older than 80 years of age [13].

However, prescription rates of statin have been shown to be declined with increasing age, and are lower, especially in elderly patients (>75 years old) [14]. Furthermore, there has been a lack of data regarding the efficacy and safety of high-intensity statin in the elderly patients (≥75 years old), mainly because this patient group is often underrepresented in randomised controlled trials comparing statin intensity. For this reason, the level of evidence of using a high-intensity statin for elderly patients is lower compared to that for younger patients in current guidelines [11,12], of which elderly patients were divided the age group by 75-years old. Moreover, there are no studies comparing the efficacy of statin intensity according to age in AMI patients treated by PCI using drug-eluting stents (DESs).

Therefore, we sought to (1) investigate the prescription rate of high-intensity statin and (2) compare the association between statin-intensity (high-intensity versus low-to-moderate-intensity statin) and clinical outcomes according to age (<75 or ≥75 years old) using large prospective AMI registry.

## Methods

### Study design and population

**Fig 1:**
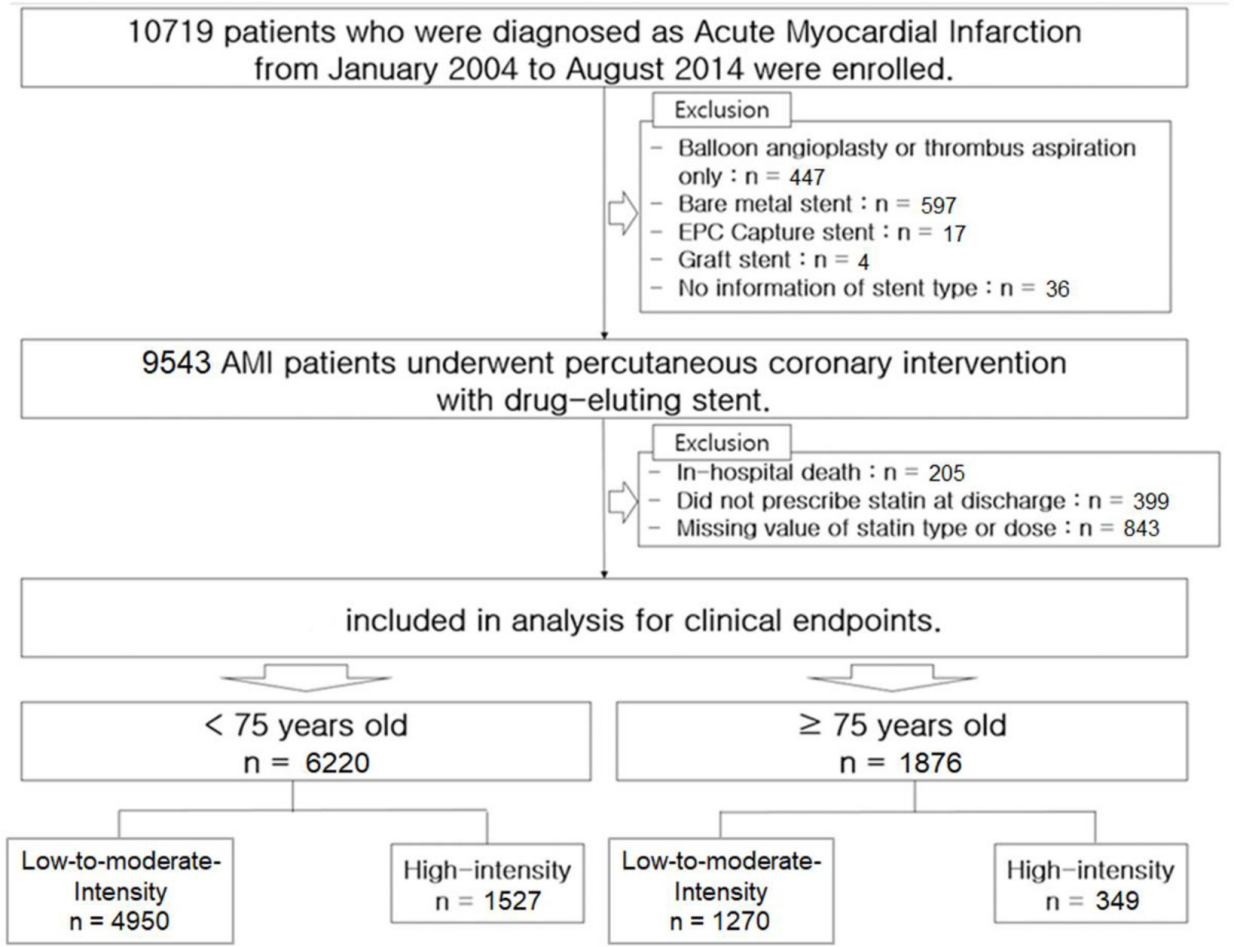
Patient flow chart.

COREA-AMI (CardiOvascular Risk and idEntificAtion of potential high-risk population in Acute Myocardial Infarction) registry is a prospective, multicenter registry including AMI patients underwent PCI between January 2004 and August 2014. It was designed to evaluate the long-term clinical outcomes in all consecutive patients with AMI at nine major cardiac centers in Korea. All hospitals were high-volume centers undergoing PCI and are located throughout the country. Clinical, angiographic and follow-up data of all AMI patients were consecutively registered in the electronic, web-based case report system. Of 10,719, we included 8,096 patients who underwent PCI using DES and had available information of statin therapy at discharge. However, patients who treated with bare-metal stent, plain old balloon angioplasty, or thrombectomy alone, and patients who experienced in-hospital death were excluded (Fig 1). All patients were classified into two groups by statin type and dose at discharge; high-intensity and low-to-moderate intensity statin group, based on American College of Cardiology/American Heart Association Classification of Intensity [15]. High-intensity statin included 40-80mg of atorvastatin or 20-40mg of rosuvastatin. Others were classified into low-to-moderate-intensity statin. We compared the efficacy on clinical outcomes according to statin-intensity in younger (<75 years old) and elderly (≥75 years old) patients, respectively.

The choice of treatment strategy including statin intensity was at the discretion of the attending cardiologists with careful consideration of clinical risk factors, anatomical complexity, and procedural characteristics. All procedure was guided by standard techniques and management. Antiplatelet therapy and peri-procedural anticoagulation were performed in accordance with the accepted guidelines. During follow-up, patient management including medical treatment was performed in accordance with accepted guidelines and established standards of care. This study was approved by the local ethics committee of each hospital, and all patients provided written informed consent for the use of their clinical data for the registry study.

### Study outcomes and follow-up

The primary end point of the study was target-vessel failure (TVF), defined as a composite of cardiovascular (CV) death, target-vessel MI (TV-MI), or target lesion revascularization (TLR). The secondary end points included the individual components of primary end point, and any death. Cause of death was considered to be cardiovascular-related, unless an unequivocal non-cardiovascular cause could be established. AMI is based on the third universal definition of myocardial infarction [16]. TV-MI was defined as newly developed “AMI” due to treated vessel after discharge. TLR was defined as any percutaneous or surgical revascularization of the treated lesion. Complex PCI was defined as one of the followings: 1) bifurcation lesion; 2) chronic total occlusion (CTO) lesion; 3) PCI for left main lesion; 4) multivessel disease; 5) restenosis lesion; 6) long lesion (total length of stents ≥60 mm); and 7) number of implanted stents ≥3. All clinical events were confirmed by source documentation collected at each hospital and centrally adjudicated by an independent group of clinicians unaware of the revascularization type.

Clinical follow-up was performed at 1 month, 6 month, 12 month, and annually after index PCI. We performed a 1-month landmark analysis separated by acute (<1 month after index PCI) and maintenance phase (from 1 month to 12 months after index PCI) to estimate accurate efficacy of statin therapy. In acute phase of AMI, the ischemic complication (ie, cardiac death, MI or stent thrombosis etc…) frequently occurs in spite of optimal medical treatment including statin, because the status of patients suffering from AMI is still unstable and several factors may affect the early period of clinical outcomes. Thus, we decided to perform 1-month landmark analysis to avoid this issue of bias and figure out the effect of statin-intensity on stabilized patients who experienced AMI.

### Statistical analyses

Continuous variables were presented as mean ± standard deviation and compared using Student *t* test or Mann-Whitney *U* test. Categorical variables were presented as counts (percentages) and compared using the chi-square or Fisher exact test, as appropriate. Event rates were estimated on Kaplan–Meier estimates in time-to-first-event analyses and were compared using the log-rank test. The Cochran-Armitage trend test was used to determine difference in trends in incidence changes of event rates between acute and maintenance phase.

Clinical outcomes were evaluated between high- versus low-to-moderate-intensity statin and stratified by the age of 75. Crude and adjusted risks for clinical outcomes were compared by univariate and multivariable Cox proportional hazards regression analyses. Multiple regression analyses using Cox proportional hazard models were performed with low-to-moderate-intensity group as the reference category and with high-intensity group as the indicator variable. Variables with P values of ≤0.1 and clinically relevant covariates irrespective of their statistical relevance in univariate analyses were candidates for inclusion in multivariate Cox proportional hazards models.

Inverse probability of treatment weighting (IPTW) based on the propensity score (probability of receiving high-intensity statin, PS) was used as the primary tool to adjust for differences in the baseline characteristics between the high-intensity and low-to-moderate intenstiy groups. Once each patient’s PS was estimated, weights were calculated using the method described in the previous literature [17]. We examined the similarity of the baseline characteristics between the treatment groups before and after IPTW [18]. After confirming the comparability of the two groups in the data with IPTW, we ran the Cox proportional hazard model and made statistical inference using robust standard errors (Huber sandwich estimator) [19].

For all crude, multivariable-adjusted, and IPTW analyses, treatment effects were evaluated in overall patients and in each group of younger and elderly patients. To test the statistical significance of the difference in treatment effect of statin-intensity between younger and elderly patients, the interaction term between age (<75 or ≥75 years old) and statin intensity was included in the multivariate that were built basis of the data from all the patients. As the sensitivity analyses, we conducted propensity matched analysis to adjust for potential confounders with a logistic regression between two statin-intensity groups. The PS was calculated from the variables including age, gender, presence of diabetes mellitus, prior MI, prior coronary bypass grafting surgery (CABG), prior cerebrovascular attack, left main disease, complex PCI, and baseline value of LDL-cholesterol. Using the PS, patients were selected by 1:1 matching without replacement using the nearest-neighbor method. A caliper width of 0.2 standardized differences (SD) was used for matching. All reported p values were two-sided, and p values <0.05 were considered statistically significant. R 4.0.2 (R Foundation for Statistical Computing, Vienna, Austria.) was used for all statistical analyses.

## Results

### Baseline patients, lesions, and procedural characteristics

Baseline characteristics of overall patients were presented in Table 1. Elderly patients (≥75 years old) accounted for 20.0% (n = 1,619) of study population. Among overall patients, 6220 (76.8%) patients were in low-to-moderate-intensity statin group, and 1876 (23.2%) patients were in high-intensity statin group. This pattern in proportion of statin-intensity was observed from younger and elderly patients.

**Table 1:** Demographics.

	Overall			<75 years old			≥75 years old		
Characteristics	Low-to-moderate- intensity (n = 6220)	High-intensity (n = 1876)	P	Low-to-moderate- intensity (n = 4950)	High-intensity (n = 1527)	P	Low-to-moderate-intensity (n = 1270)	High-intensity (n = 349)	P
**Baseline Patients’ characteristics**									
Age (years)	63.1 ± 12.8	61.9 ± 12.7	0.001	58.7 ± 10.2	57.8 ± 10.0	0.001	80.1 ± 5.4	80.2 ± 5.2	0.68
Male	4484 (72.1)	1421 (75.7)	0.002	3889 (78.6)	1249 (81.8)	0.007	595 (46.9)	172 (49.3)	0.46
Hypertension	3185 (51.2)	941 (50.2)	0.43	2347 (47.5)	716 (46.9)	0.72	838 (66.0)	225 (64.5)	0.64
Diabetes mellitus	1930 (31.1)	533 (28.4)	0.031	1521 (30.8)	420 (27.5)	0.017	409 (32.2)	113 (32.4)	1.00
Hyperlipidemia	924 (14.9)	562 (19.3)	<0.001	775 (15.7)	317 (20.8)	<0.001	149 (11.7)	45 (12.9)	0.62
Current smoker	2571 (41.3)	826 (44.0)	0.041	2385 (48.2)	765 (50.1)	0.20	186 (14.6)	61 (17.5)	0.22
Clinical diagnosis			0.19			0.18			0.60
STEMI	3378 (54.3)	986 (52.6)		2788 (56.3)	830 (54.4)		590 (46.5)	156 (44.7)	
NSTEMI	2840 (45.7)	890 (47.4)		2160 (43.7)	697 (45.6)		680 (53.5)	193 (55.3)	
CKD	94 (1.5)	35 (1.9)	0.33	80 (1.6)	29 (1.9)	0.53	14 (1.1)	6 (1.7)	0.41
Prior MI	219 (3.5)	35 (1.9)	<0.001	155 (3.1)	23 (1.5)	0.001	64 (5.0)	12 (3.4)	0.27
Prior PCI	370 (6.0)	85 (4.5)	0.022	258 (5.2)	64 (4.2)	0.12	112 (8.8)	21 (6.0)	0.12
Prior CABG	29 (0.5)	3 (0.2)	0.1	20 (0.4)	1 (0.1)	0.04	9 (0.7)	2 (0.6)	1.00
Prior CVA	427 (6.9)	111 (5.9)	0.16	300 (6.1)	75 (4.9)	0.10	127 (10.0)	36 (10.3)	0.94
PAD	31 (0.5)	6 (0.3)	0.42	20 (0.4)	5 (0.3)	0.85	11 (0.9)	1 (0.3)	0.48
AF	180 (2.9)	21 (1.1)	<0.001	116 (2.3)	14 (0.9)	0.001	64 (5.0)	7 (2.0)	0.021
LV EF, %	53.5 ± 10.9	53.6 ± 10.9	0.94	54.2 ± 10.5	54.2 ± 10.7	0.94	51.1 ± 11.9	50.9 ± 11.6	0.84
Total Cholesterol	176 (150–204)	182 (154–212)	<0.001	178 (153–206)	184 (157–214)	<0.001	166 (140–195)	168 (140–199)	0.62
Triglyceride	100 (67–149)	115 (77–166)	<0.001	104 (71–154)	123 (81–175)	<0.001	84 (59–123)	89 (63–126)	0.09
HDL Cholesterol	40 (34–47)	40 (34–46)	0.15	40 (34–47)	39 (34–46)	0.25	41 (34–49)	40 (34–48)	0.43
LDL Cholesterol	112 (88–136)	118 (93–144)	<0.001	114 (90–138)	120 (96–145)	<0.001	105 (81–131)	107 (81–132)	0.52
**Discharge medication**									
Aspirin	6142 (98.8)	1848 (98.7)	0.83	4892 (98.8)	1503 (98.6)	0.44	1250 (98.4)	345 (99.1)	0.45
P2Y_12_ inhibitor	6080 (98.0)	1850 (98.9)	0.02	4838 (98.1)	1505 (98.8)	0.06	1242 (97.9)	345 (99.1)	0.21
Clopidogrel	5392 (86.7)	1405 (75.1)	<0.001	4241 (85.7)	1108 (72.7)	<0.001	1151 (90.6)	297 (85.3)	0.006
Ticagrelor	266 (4.3)	239 (12.7)	<0.001	183 (3.7)	191 (12.5)	<0.001	83 (6.5)	48 (13.8)	<0.001
Prasugrel	451 (7.3)	213 (11.4)	<0.001	439 (8.9)	213 (14.0)	<0.001	12 (0.9)	0	0.08
**Lesion and procedural characteristics**									
Radial access	1248 (20.1)	370 (19.7)	0.20	960 (19.4)	309 (20.2)	0.35	288 (22.7)	61 (17.5)	0.015
LM involved	390 (6.3)	122 (6.5)	0.76	267 (5.4)	98 (6.4)	0.15	123 (9.7)	24 (6.9)	0.13
pLAD involved	2645 (42.5)	842 (44.9)	0.08	2054 (41.5)	680 (44.5)	0.038	591 (46.5)	162 (46.4)	>0.99
Disease extent			0.40			0.24			0.52
1VD	2761 (44.4)	851 (45.4)		2325 (46.9)	723 (47.4)		466 (36.7)	141 (40.4)	
2VD	2056 (33.1)	625 (33.3)		1620 (32.7)	516 (33.8)		436 (34.3)	109 (31.2)	
3VD	1373 (22.1)	387 (20.6)		1005 (20.3)	288 (18.9)		368 (29.0)	99 (28.4)	
Complex PCI	2710 (43.6)	809 (43.1)		2124 (42.9)	654 (42.8)	0.98	586 (46.1)	155 (44.4)	0.61
Total stent number	1.63 ± 0.88	1.64 ± 0.89	0.70	1.61 ± 0.88	1.64 ± 0.90	0.38	1.68 ± 0.86	1.64 ± 0.87	0.38
Mean stent diameter	3.17 ± 0.40	3.16 ± 0.56	0.67	3.20 ± 0.41	3.19 ± 0.52	0.50	3.07 ± 0.35	3.07 ± 0.72	0.96
Total stent length	34.9 ± 21.0	34.6 ± 22.0	0.61	34.57 ± 21.0	34.65 ± 22.3	0.91	36.00 ± 21.1	34.26 ± 20.7	0.17
IVUS use	1352 (21.7)	372 (19.8)	0.08	1135 (22.9)	314 (20.6)	0.057	217 (17.1)	58 (16.6)	0.90

STEMI, ST-segment elevation myocardial infarction; NSTEMI, non ST-segment elevation myocardial infarction; CKD, chronic kidney disease; MI, myocardial infarction; PCI, percutaneous coronary intervention; CABG, coronary artery bypass grafting surgery; CVA, cerebrovascular attack; PAD, peripheral artery disease; AF, atrial fibrillation; LV EF, left ventricle ejection fraction; HDL, high-density lipoprotein; LDL, low-density lipoprotein; LM, left main; pLAD, proximal left anterior descending artery; VD, vessel disease; IVUS, intravascular ultrasound.

In younger patients (<75 years old), mean age was lower (57.8 ± 10.0 vs. 58.7 ± 10.2, p<0.001) and the proportion of male gender were higher (81.8% vs. 78.6%, p = 0.007) in high-intensity statin group than low-to-moderate-intensity statin group. The high-intensity statin group had a significantly greater prevalence of hyperlipidemia and lesser prevalence of diabetes mellitus, prior MI, prior CABG, and atrial fibrillation compared with low-to-moderate intensity statin group. As the discharge medication, patients in the high-intensity statin group were more likely to receive potent P2Y12 inhibitors (ticagrelor or prasugrel) than low-to-moderate-intensity statin group. In elderly patients, baseline demographic characteristics did not differ between two statin-intensity groups. In overall patients, mean stent diameter, total stent number and length was similar between the two statin-intensity groups, that was consistent regardless of age. Involvement of proximal left anterior descending artery was shown frequently in high-intensity statin group among younger patients (Table 1). As shown in Table 2 and Table 2 in S1 Appendix, majority of those differences of covariates were balanced after PS matching and IPTW.

**Table 2:** Demographics after inverse probability weighting.

	<75 years old				≥75 years old			
Characteristics	Low-to-moderate intensity (n = 6133)	High-intensity (n = 5683)	P	SMD	Low-to-moderate intensity (n = 1548)	High-intensity (n = 1435)	P	SMD
**Baseline patients characteristics**								
Age (years)	58.5 ± 10.2	58.3 ± 9.8	0.64	0.015	80.1 ± 5.4	80.0 ± 5.1	0.76	0.019
Male	4878 (79.5)	4592 (80.8)	0.33	0.032	733 (47.4)	691 (48.1)	0.81	0.016
Hypertension	2896 (47.2)	2689 (47.3)	0.96	0.002	1012 (65.4)	915 (63.8)	0.61	0.034
Diabetes mellitus	1841 (30.0)	1687 (29.7)	0.82	0.007	505 (32.6)	470 (32.8)	0.97	0.003
Hyperlipidemia	1068 (17.4)	1137 (20.0)	0.039	0.066	192 (12.4)	212 (14.8)	0.32	0.068
Current smoker	2994 (48.8)	2789 (49.1)	0.88	0.005	235 (15.2)	207 (14.4)	0.74	0.021
Clinical diagnosis			0.55	0.019			0.99	0.002
STEMI	3417 (55.7)	3111 (54.7)			712 (46.0)	659 (45.9)		
NSTEMI	2717 (44.3)	2573 (45.3)			836 (54.0)	777 (54.1)		
CKD	99.3 (1.6)	87.5 (1.5)	0.84	0.006	19 (1.2)	18 (1.3)	0.96	0.003
Prior MI	156 (2.5)	135 (2.4)	0.77	0.011	72 (4.6)	62 (4.4)	0.84	0.014
Prior PCI	290 (4.7)	266 (4.7)	0.94	0.002	127 (8.2)	116 (8.1)	0.95	0.004
Prior CABG	19 (0.3)	10 (0.2)	0.58	0.027	11 (0.7)	11 (0.8)	0.90	0.009
Prior CVA	350 (5.7)	323 (5.7)	0.97	0.001	158 (10.2)	142 (9.9)	0.86	0.011
PAD	24 (0.4)	24 (0.4)	0.90	0.005	12 (0.8)	10 (0.7)	0.92	0.009
AF	121 (2.0)	93 (1.6)	0.53	0.025	70 (4.5)	54 (3.8)	0.65	0.039
LV EF, %	54.2 ± 10.5	54.2 ± 10.7	0.83	0.007	51.0 ± 11.9	50.7 ± 11.6	0.67	0.028
**Lesion and Procedural characteristics**								
Radial access	1244 (20.3)	1212 (21.3)	0.40	0.026	343 (22.1)	333 (23.2)	0.71	0.027
LM involved	353 (5.8)	336 (5.9)	0.84	0.006	139 (9.0)	118 (8.2)	0.70	0.028
pLAD involved	2541 (41.4)	2500 (44.0)	0.11	0.052	707 (45.7)	693 (48.3)	0.44	0.051
Disease extent			0.44	0.052			0.35	0.12
1VD	2939 (47.9)	2715 (47.8)			572 (37.0)	607 (42.3)		
2VD	1966 (32.1)	1870 (32.9)			539 (34.8)	463 (32.2)		
3VD	1228 (20.0)	1099 (19.3)			437 (28.2)	366 (25.5)		
Complex PCI	2636 (43.0)	2484 (43.7)	0.65	0.015	705 (45.6)	665 (46.3)	0.82	0.015
Total stent number	1.62 ± 0.88	1.64 ± 0.91	0.47	0.025	1.67 ± 0.85	1.66 ± 0.90	0.87	0.012
Mean stent diameter	3.20 ± 0.42	3.20 ± 0.51	0.97	0.001	3.07 ± 0.35	3.07 ± 0.77	0.94	0.006
Total stent length	34.47 ± 20.97	35.20 ± 22.73	0.34	0.033	35.65 ± 20.66	34.97 ± 21.53	0.65	0.032
IVUS use	1382 (22.5)	1279 (22.5)	0.98	0.001	261 (16.8)	234 (16.3)	0.82	0.015

SMD, standardised mean difference; STEMI, ST-segment elevation myocardial infarction; NSTEMI, non ST-segment elevation myocardial infarction; CKD, chronic kidney disease; MI, myocardial infarction; PCI, percutaneous coronary intervention; CABG, coronary artery bypass grafting surgery; CVA, cerebrovascular attack; PAD, peripheral artery disease; AF, atrial fibrillation; LV EF, left ventricle ejection fraction; HDL, high-density lipoprotein; LDL, low-density lipoprotein; LM, left main; pLAD, proximal left anterior descending artery; VD, vessel disease; IVUS, intravascular ultrasound.

### Incidence rates of adverse clinical outcomes among overall patients

In overall patients, the event rates of clinical outcomes during 1 year and adjusted hazard ratio (aHR) for risk of high-intensity statin compared with low-to-moderate intensity statin are shown in Table 1 in S1 Appendix. Adverse clinical outcomes were similar between two statin-intensity groups except for TLR, which was significantly lower in the high-intensity statin group (TVF: aHR, 0.9; 95% confidence interval [CI], 0.74–1.09; p = 0.28; CV death: aHR, 1.12; 95% CI, 0.81–1.46; p = 0.59; TV-MI: aHR, 1.08; 95% CI, 0.6–1.93; p = 0.81; TLR: aHR, 0.74; 95% CI, 0.57–0.96; p = 0.025). We also investigated the monthly incidence rates of clinical outcomes in overall population to identify a discriminating trends between acute and maintenance phase (Fig 1 in S1 Appendix). As described Fig 1 in S1 Appendix, Cochran-Armitage trend testing revealed these trends to be statistically significant in terms of TVF, CV death and TV-MI (p≤0.01), which showed relatively higher incidence rates of clinical outcomes in acute phase (<1 month) and decreased trend in maintenance phase (from 1 month to 12 months).

### Adverse clinical outcomes within acute phase (<1 month)

**Fig 2:**
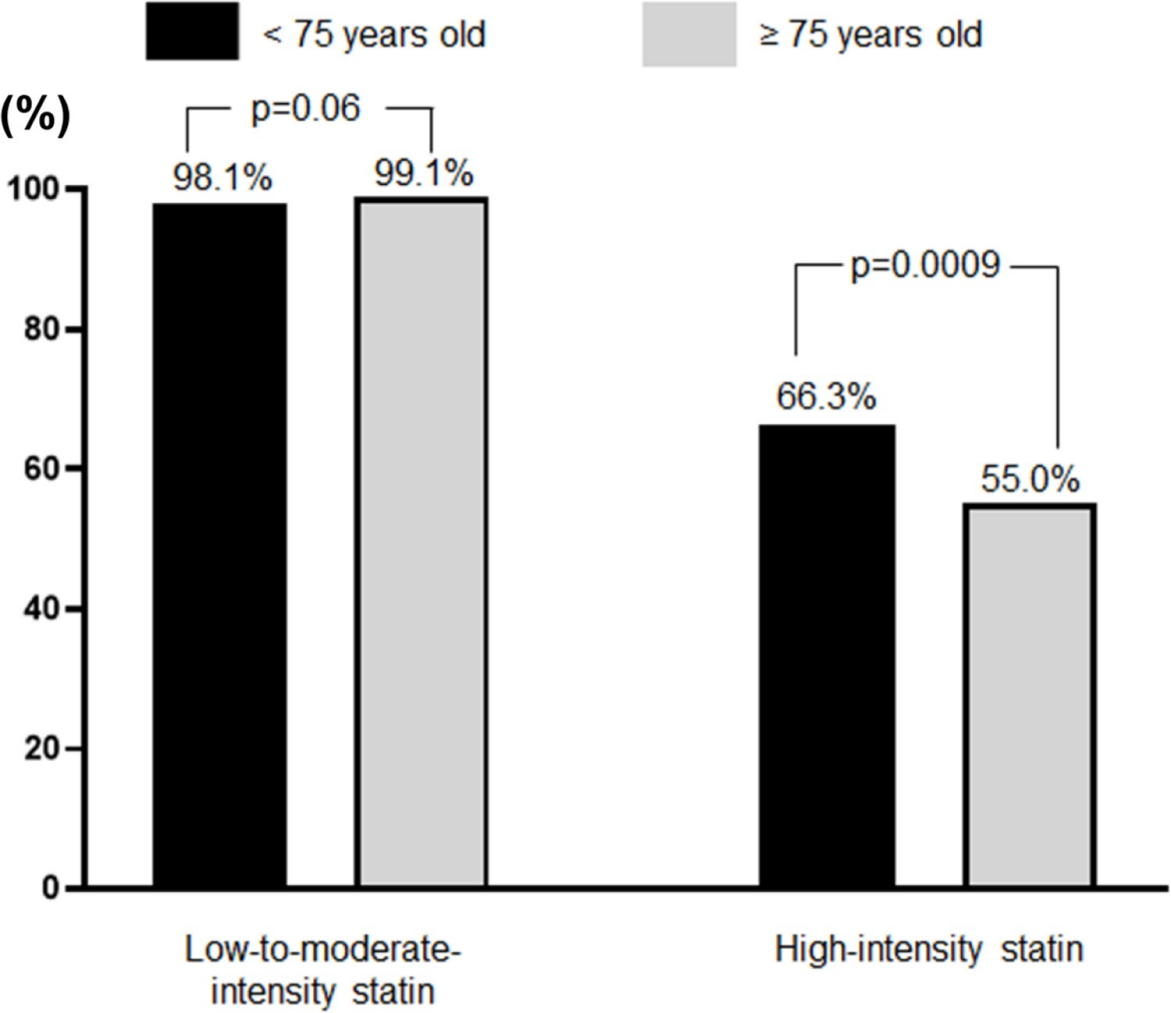
Adjusted Kaplan-Meier curves for the primary end point according to statin-intensity in younger and elderly patients using inverse probability weighting.

The incidence rates of clinical outcomes between two statin-intensity groups in the overall population, younger and elderly patients within 1 month after index PCI are shown in Table 3. Kaplan-Meier curves for TVF at 1 month were shown in Fig 2, adjusted by IPTW analysis. In overall patients, the high-intensity statin group showed poorer 1-month clinical outcomes regarding TVF, all-cause death and CV death (TVF: aHR, 1.72; 95% CI, 1.07–2.78; p = 0.026; all-cause death: aHR, 2.21; 95% CI, 1.21–4.04; p = 0.01; CV death: aHR, 2.16; 95% CI, 1.16–4.02; p = 0.015). However, after IPTW analysis, no differences were observed with statin-intensity in both younger and elderly patients (Table 3).

**Table 3:** Event rates and hazard ratios for clinical outcomes in acute phase (<1 month).

Outcomes	Event Rates at 1 Month (n/%*)		Crude		Multivariate Adjusted†		IPTW Adjusted		
	Low-to-moderate-intensity	High-intensity	HR (95% CI)	P	HR (95% CI)	P	HR (95% CI)	P	P_Interaction_
**Overall**									
TVF	52 (0.8)**	25 (1.3)**	1.6 (1.0–2.58)	0.052	1.72 (1.07–2.78)	0.026	1.24 (0.73–2.10)	0.43	0.97
All-cause death	28 (0.5)**	17 (0.9)**	2.03 (1.11–3.7)	0.022	2.21 (1.21–4.04)	0.01	1.37 (0.70–2.70)	0.36	0.96
CV death	27 (0.4)**	16 (0.9)**	1.98 (1.07–3.67)	0.031	2.16 (1.16–4.02)	0.015	1.32 (0.65–2.66)	0.44	0.68
TV-MI	10 (0.2)	6 (0.3)	2.0 (0.73–5.49)	0.18	2.07 (0.75–5.71)	0.16	1.72 (0.61–4.86)	0.31	0.31
TLR	23 (0.4)	5 (0.3)	0.72 (0.27–1.9)	0.51	0.76 (0.29–2.0)	0.58	0.65 (0.24–1.78)	0.40	0.73
**< 75 years old**									
TVF	31 (0.6)	16 (1.1)	1.68 (0.92–3.07)	0.09	1.84 (1.00–3.37)	0.048	1.23 (0.63–2.38)	0.54	
All-cause death	16 (0.3)	10 (0.7)	2.04 (0.92–4.49)	0.08	2.34 (1.06–5.17)	0.036	1.39 (0.57–3.38)	0.47	
CV death	15 (0.3)	10 (0.7)	2.17 (0.98–4.84)	0.057	2.49 (1.12–5.56)	0.026	1.48 (0.60–3.64)	0.39	
TV-MI	8 (0.2)	4 (0.3)	1.63 (0.49–5.4)	0.43	1.72 (0.52–5.37)	0.38	1.16 (0.35–3.90)	0.81	
TLR	14 (0.3)	4 (0.3)	0.93 (0.31–2.82)	0.90	0.99 (0.32–3.01)	0.98	0.73 (0.23–2.29)	0.59	
**≥ 75 years old**									
TVF	21 (1.7)	9 (2.6)	1.57 (0.72–3.44)	0.26	1.57 (0.72–3.42)	0.26	1.21 (0.51–2.87)	0.66	
All-cause death	12 (0.9)	7 (2.0)	2.14 (0.84–5.44)	0.11	2.13 (0.84–5.4)	0.11	1.37 (0.46–4.04)	0.57	
CV death	12 (0.9)	6 (1.7)	1.84 (0.69–4.89)	0.22	1.82 (0.68–4.86)	0.23	1.08 (0.34–3.37)	0.90	
TV-MI	2 (0.2)	2 (0.6)	3.65 (0.51–25.9)	0.20	3.63 (0.51–25.8)	0.20	3.92 (0.5–30.53)	0.19	
TLR	9 (0.7)	1 (0.3)	0.41 (0.05–3.2)	0.39	0.41 (0.05–3.25)	0.40	0.48 (0.07–3.61)	0.48	

†adjusted by covariates including age, diabetes mellitus.

*Event rates were derived from the Kaplan-Meier estimates. Hazard ratio is the risk of high-intensity statin for clinical outcomes compared with that of less intensive statin.

**P value by log-rank test was less than 0.05.

HR, hazard ratio; TVF, target vessel failure; CV, cardiovascular; TV-MI, target-vessel myocardial infarction; TLR, target lesion revascularization.

After subgroup analysis by aged of 75, these findings were consistent in younger patients. Meanwhile, in elderly patients, despite numerically higher event rates in the high-intensity statin group, there were no statistical significance (Table 3). No significant interactions were found between aged group and statin intensity in any of the adjusted 1-month risks of study outcomes (P_interaction_ = 0.97 for TVF, P_interaction_ = 0.96 for all-cause death, P_interaction_ = 0.68 for CV death, P_interaction_ = 0.31 for TV-MI and P_interaction_ = 0.73 for TLR).

### Adverse clinical outcomes in maintenance phase (from 1 month to 12 months)

The incidence rates of clinical outcomes between two statin-intensity groups in the overall population, younger and elderly patients in maintenance phase are shown in Table 4. Kaplan-Meier curves for TVF and secondary end points in maintenance phase were shown in Fig 2 and Fig 2 in S1 Appendix, which were adjusted by IPTW analysis. In overall population, the risk of adverse clinical outcomes according to statin-intensity was not different except for TLR (TVF: aHR, 0.85; 95% CI, 0.69–1.05; p = 0.12; TLR: aHR, 0.73; 95% CI, 0.55–0.96; p = 0.022; Table 4). In younger patients, the high-intensity statin group showed significantly better clinical outcomes in terms of TVF (aHR, 0.76; 95% CI, 0.59–0.99; p = 0.038) and TLR (aHR, 0.72; 95% CI, 0.54–0.97; p = 0.032) than the low-to-moderate-intensity statin group. However, intriguingly, in elderly patients, the incidence rates of adverse clinical outcomes between two statin-intensity groups did not differ (TVF: aHR, 1.1; 95% CI, 0.76–1.59; p = 0.63; TLR: aHR, 0.76; 95% CI, 0.37–1.57; p = 0.46). These findings were unchanged after IPTW adjustment for differences in baseline covariates.

**Table 4:** Event rates and hazard ratios for clinical outcomes in maintenance phase (from 1 month to 12 months).

Outcomes	Event Rates at 1–12 Month (n/%*)		Crude		Multivariate Adjusted†		IPTW Adjusted		
	Low-to-moderate-intensity	High-intensity	HR (95% CI)	P	HR (95% CI)	P	HR (95% CI)	P	P_Interaction_
**Overall**									
TVF	460 (7.7)	117 (6.6)	0.86 (0.7–1.05)	0.14	0.85 (0.69–1.05)	0.12	0.85 (0.68–1.06)	0.15	0.17
All-cause death	220 (3.7)	71 (4.0)	1.1 (0.84–1.43)	0.50	1.15 (0.87–1.52)	0.33	1.17 (0.86–1.57)	0.32	0.30
CV death	164 (2.7)	53 (3.0)	1.1 (0.8–1.49)	0.56	1.1 (0.79–1.53)	0.58	1.06 (0.75–1.49)	0.76	0.28
TV-MI	38 (0.6)	10 (0.6)	0.89 (0.45–1.79)	0.75	0.83 (0.4–1.73)	0.62	0.84 (0.37–1.92)	0.69	NA
TLR	295 (5.0)**	64 (3.7)**	0.73 (0.56–0.96)	0.024	0.73 (0.55–0.96)	0.022	0.72 (0.54–0.97)	0.033	0.76
**< 75 years old**									
TVF	329 (6.9)	79 (5.5)	0.79 (0.62–1.01)	0.059	0.76 (0.59–0.99)	0.038	0.75 (0.57–0.99)	0.044	
All-cause death	107 (2.2)	32 (2.2)	0.99 (0.67–1.47)	0.95	0.97 (0.63–1.49)	0.89	0.96 (0.61–1.52)	0.86	
CV death	76 (1.6)	22 (1.5)	0.96 (0.59–1.54)	0.85	0.85 (0.5–1.46)	0.56	0.82 (0.47–1.43)	0.48	
TV-MI	27 (0.6)	10 (0.7)	1.22 (0.59–2.53)	0.59	1.11 (0.52–2.39)	0.79	1.19 (0.51–2.76)	0.69	
TLR	250 (5.3)**	55 (3.9)**	0.72 (0.54–0.97)	0.029	0.71 (0.53–0.96)	0.025	0.70 (0.51–0.97)	0.033	
**≥ 75 years old**									
TVF	131 (10.8)	38 (11.7)	1.1 (0.76–1.58)	0.61	1.1 (0.76–1.59)	0.63	1.06 (0.72–1.57)	0.76	
All-cause death	113 (9.2)	39 (11.8)	1.32 (0.91–1.89)	0.14	1.35 (0.92–1.97)	0.12	1.33 (0.90–1.97)	0.15	
CV death	88 (7.2)	31 (9.5)	1.34 (0.89–2.02)	0.16	1.34 (0.88–2.05)	0.18	1.24 (0.80–1.92)	0.35	
TV-MI	11 (0.9)	0	NA		NA		NA		
TLR	45 (3.9)	9 (2.9)	0.76 (0.37–1.55)	0.45	0.76 (0.37–1.57)	0.46	0.81 (0.37–1.75)	0.59	

†adjusted by covariates including age, diabetes mellitus.

*Event rates were derived from the Kaplan-Meier estimates. Hazard ratio is the risk of high-intensity statin for clinical outcomes compared with that of less intensive statin.

**P value by log-rank test was less than 0.05.

HR, hazard ratio; TVF, target vessel failure; CV, cardiovascular; TV-MI, target-vessel myocardial infarction; TLR, target lesion revascularization.

**Fig 3:**
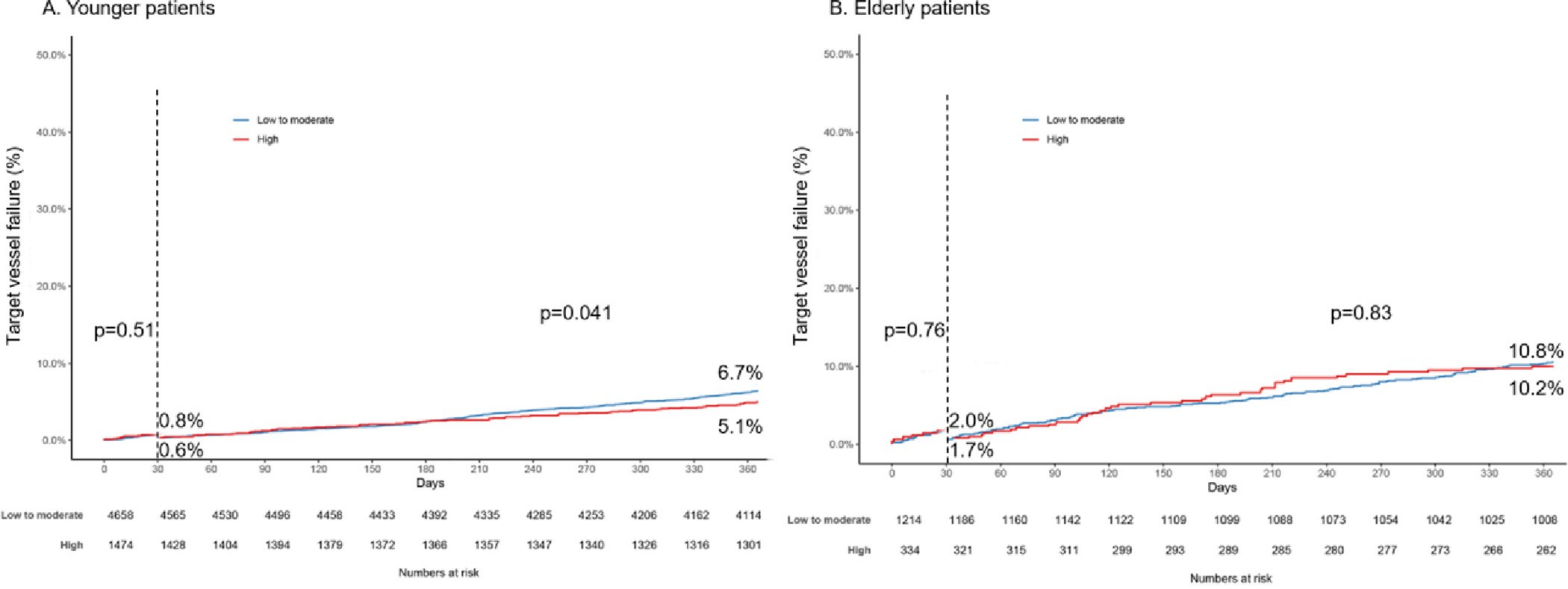
Effects of statin intensity on clinical outcomes in maintenance phase after IPTW analysis, subdivided by aged of 75. *derived from unmatched population. **aHR, adjusted hazard ratio for high-intensity statin treatment compared with less intensive strategy after IPTW analysis. TVF, target-vessel failure; TLR, target lesion revascularization; HR, hazard ratio; NA, not applicable.

The forest plot of hazard ratio after IPTW analysis for adverse clinical outcomes from 1 month to 12 months was shown in Fig 3. No significant treatment interactions were detected in subgroups defined by aged of 75 (P_interaction_ = 0.18 for TVF, P_interaction_ = 0.35 for all-cause death, P_interaction_ = 0.15 for CV death, and P_interaction_ = 0.76 for TLR).

### Sensitivity analysis after PS matching

After PS matching between high- and low-to-moderate-intensity statin groups, there were 1,398 and 305 matched pairs in younger and elderly patients, respectively. There were no other significant differences for any of the covariates between two statin-intensity groups, except for some covariates (hypertension, hyperlipidemia, clinical diagnosis, atrial fibrillation, baseline value of triglyceride and use of IVUS) in younger patients (Table 2 in S1 Appendix).

The adverse clinical outcomes in this sub-cohort and in each group stratified by statin-intensity are shown in Table 3 in S1 Appendix and Fig 3 in S1 Appendix. These sensitivity analyses revealed consistent findings with the relative effect of high- and low-to-moderate-intensity statin according to aged groups, in which the better clinical outcomes of high-intensity statin group was more prominent in younger patients.

### Subgroup analysis for clinical impact of statin-intensity among patients with a maintained intensity of statin

**Fig 4:**
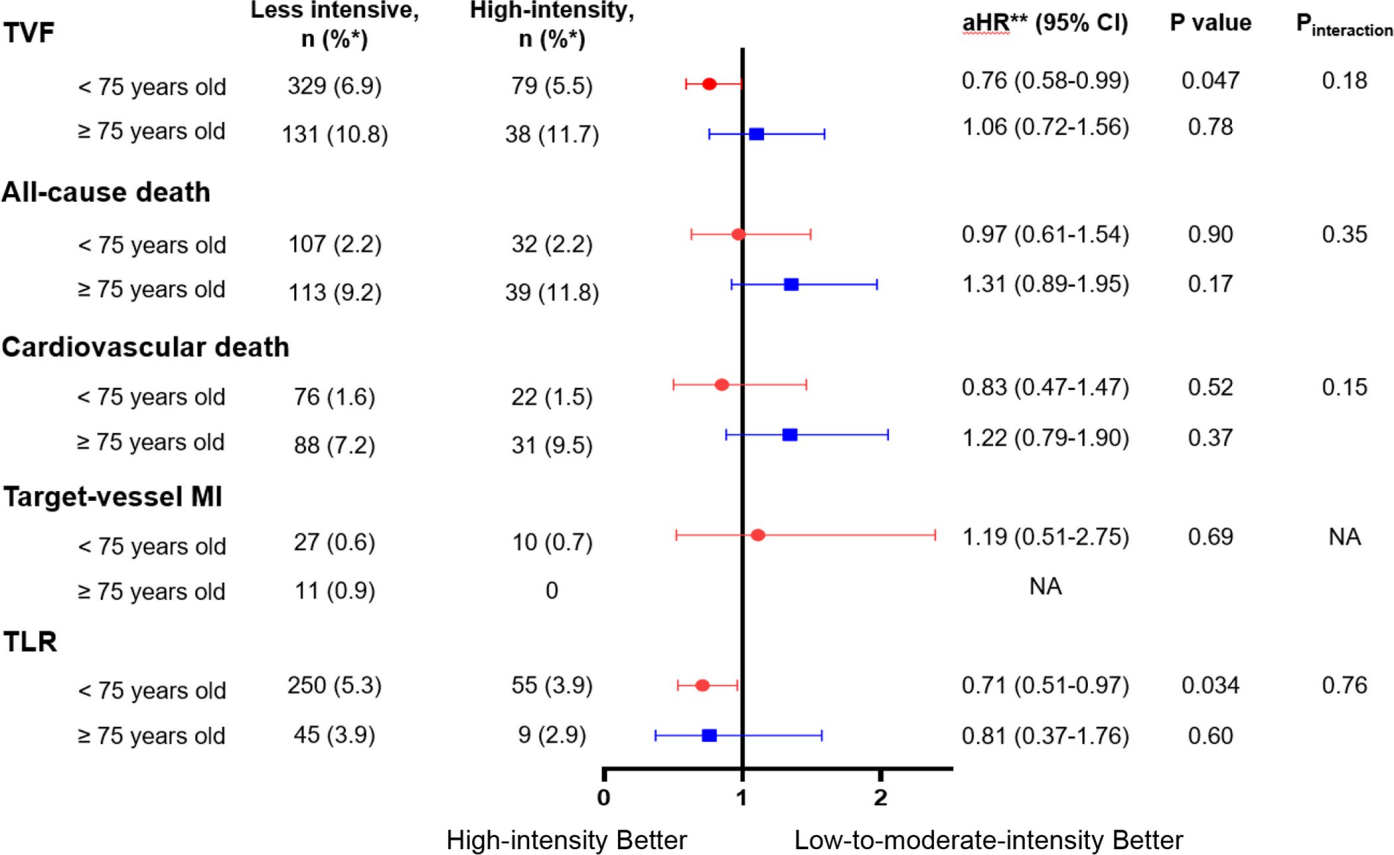
Age differences on maintenance proportion of statin-intensity.

In order to further support the result of our study, we performed subgroup analysis with patients who continued the same intensity of statin therapy. Because adherence and tolerance are important clinical aspects of medical treatment that affect clinical outcomes [20], we investigated the maintenance proportion of the same intensity of statin during 1 year after index AMI in total of 6,566 patients. In high-intensity statin group, the proportion of patients continuing the same statin-intensity was significantly lower in elderly patients than that of younger patients (55.0% vs. 66.3%, p = 0.0009), while this phenomenon was not observed in low-to-moderate-intensity statin group (99.1% vs. 98.1%, p = 0.06) (Fig 4).

Table 5 in S1 Appendix shows the results of subgroup analysis, patients who maintained high-intensity statin showed significantly better clinical outcome in terms of TVF in overall and younger patients (overall: aHR 0.64, 95% CI 0.46–0.9, p = 0.01; younger patients: aHR 0.63, 95% CI 0.44–0.9, p = 0.012). However, in elderly patients, the incidence rates of adverse clinical outcomes did not differ between continuous use of high- and low-to-moderate-intensity statin (aHR 0.7, 95% CI 0.28–1.77, p = 0.46). These findings were consistent with the main results of our study. However, the incidence rate of TVF was numerically lower in high-intensity statin group compared to low-to-moderate-intensity statin group. To overcome the small number of event rates, we extended a study period to 24 months, which also showed a consistent result (Table 5 in S1 Appendix).

### Independent Predictors of TVF

As shown in Table 4 in S1 Appendix, in younger patients, high-intensity statin revealed to be an independently protective effect for TVF in maintenance phase (HR, 0.77; 95% CI, 0.59–0.99; p = 0.04), which was not in elderly patients.

## Discussion

The major findings from the present study are as follows: 1) although current guideline recommends high-intensity statin in AMI patients, low-to-moderate-intensity statins were prescribed 3 times more than high-intensity statins in daily clinical practice; 2) in high-intensity statin group, the proportion of patients continuing the same statin-intensity was significantly lower in elderly patients than that of younger patients; 3) in maintenance phase, high-intensity statin group showed the better clinical outcomes in terms of TVF, mainly by TLR than less-intensity group among younger patients; 4) in contrast, the incidence rates of adverse clinical events between high- and low-to-moderate-intensity statin were not statistically different in elderly patients.

Because the Corea-AMI registry is large, prospective, multicenter registry enrolled 10,719 AMI patients underwent PCI, we believe that our findings may reflect real-world clinical practice for secondary prevention regarding the statin-intensity. Indeed, we enrolled consecutive AMI patients with end-stage renal disease, heart failure or cardiogenic shock. Interestingly, in our data, less-intensive statins rather than high-intensity statins were prescribed 3 times more often regardless of age group (4,950 of 6,477 [76.4%] in younger patients and 1,270 of 1,619 [78.4%] in elderly patients; p = 0.09). Similar with our finding, several previous studies also have shown that high-intensity statin was not frequently used for ASCVD patients in the real-world clinical practice, in which only in 15–29.6% of patients received high-intensity statin [20–22].

Moreover, de-escalation of high-intensity statin was frequently observed in elderly patients during 1 year. Likewise, in the Patient and Provider Assessment of Lipid Management (PALM) registry, elderly patients were much more likely to receive a moderate-intensity statin rather than a high-intensity statin for secondary prevention in real-world situation [23]. The plausible explanations of this situation are paucity of evidence using high-intensity statin in elderly patients, concern about side-effect of high-intensity statin and poor compliance in elderly patients. Actually, 2019 ESC/EAS guideline mentions that statin is started at a low dose and titrated upwards if there is significant renal impairment or the potential for drug interactions, which is more likely to be observed in elderly patients [11].

In our data, through the 1-month landmark analysis, it was shown that the risk of the early adverse clinical outcomes was significantly higher in high-intensity statin than in less-intensive statin group. This may be caused by patients’ unstable condition or incomplete procedural factors during acute phase of AMI, which reflected that high-intensity statin was more likely to be prescribed in higher risk patients who were expected to have a poor prognosis.

In addition, we demonstrated that high-intensity statin showed better clinical outcomes than low-to-moderate-intensity statin in younger patients during maintenance phase, while the clinical outcomes between two statin-intensity group was similar in elderly patients. There have been several studies regarding clinical efficacy of low-to-moderate-intensity statin for secondary prevention in elderly patients. Looking at the result of Pravastatin or Atorvastatin Evaluation and Infection Therapy-Thrombolysis in Myocardial Infarction 22 (PROVE IT-TIMI 22) Trial [24], high-intensity atorvastatin was not superior to moderate-intensity pravastatin among patients aged ≥ 65 years. IDEAL (Incremental DEcrease through Aggressive Lipid Lowering) Study randomly assigned 8,888 patients age ≤ 80 years with previous myocardial infarction to receive either intensive therapy with atorvastatin 80 mg/day or the standard statin therapy (simvastatin 20 to 40 mg/day) to compare the ability of these regimens to lower cardiovascular risk. In the subgroup analysis, significant reductions in primary and secondary endpoints were observed only in patients <65 years of age compared to 65 to 80 years of age [25]. Kwak A. et al. [26] reported that moderate-intensity statin can be more protective against ischemic cardiovascular and cerebrovascular events compared with high-intensity statin in patients aged 75 years and older. Foody J.M. et al. [27] showed that statin therapy was associated with a significant reduction in all-cause mortality in patients younger than 80, but not in patients aged 80 and older. Although some analyses reported an association between high-intensity statin use and a survival benefit in older adults, the study population was heterogenous while our study population consisted of only AMI patients [28]. Even about 60% of CAD patients were included in the study population, no information of severity or revascularization of coronary lesions was available in this study [28]. Besides, in the meta-analysis by Cholesterol Treatment Trialists’ Collaboration, 26 trials were based on analysis between statin therapy versus control, not comparison of statin-intensity [29]. Thus, we need well-powered large scale randomized studies regarding this topic.

Lower efficacy of high-intensity statin than expected in patients over 75 years of age may be explained by lower endogenous cholesterol synthesis and higher cholesterol absorption in elderly subjects. In the DEBATE study of home-dwelling elderly patients, low cholesterol absorption was associated with fewer cardiovascular events and better survival, while increased cholesterol absorption was associated with increased cardiovascular mortality [30]. Also, in subgroup analysis of IMPROVE-IT, Bach RG et al. found that elderly patients did benefit in particular from the addition of ezetimibe to simvastatin. Compared with younger patients, the absolute risk reduction for the primary end point was substantially greater for patients 75 years or older [31]. These findings raise the hypothesis that cholesterol metabolism changes during lifetime: whereas midlife is characterized by higher endogenous synthesis rates and lower cholesterol absorption rates, cholesterol synthesis deteriorates with increasing age. Thus, we think that the decreased importance of endogenous cholesterol synthesis and the increased role of cholesterol absorption with increasing age may be the potential explanation of our findings. Taken together, in elderly patients who are not tolerant to high-intensity statin, we should consider additional optional treatment with statin such as ezetimibe to achieve a target of LDL-cholesterol level.

Our study has several limitations listed as follows. 1) This was a non-randomized, observational registry study with inherent methodological limitations; thus, its overall findings must be considered as hypothesis generating only. However, even though there is an inherent limitation, we could demonstrate the efficacy of high-intensity statin for secondary prevention in younger patients. This finding is well in line with the evidence provided by the current guidelines [11,12]. 2) Our findings are subject to selection bias because the treatment choice was left to the physician. However, to minimize the bias, we performed rigorous adjustments using a multivariable Cox proportional hazard regression model, PS matching and subgroup analysis with patients who were continuing the same statin-intensity during 1 year. Nevertheless, hidden bias may still remain because of the influence of unmeasured confounding factors. Therefore, our findings should be considered with caution until additional large clinical studies with long-term follow-ups replicate our findings. 3) Owing to the relatively limited number, some result of this study might not be sufficiently powered to compare between statin-intensity groups. Especially in elderly patients, high-intensity statin showed numerically higher incidence rates of TVF without statistical significance in maintenance phase. As mentioned above, we additionally performed subgroup analysis with patients who were continuing the same statin-intensity during 1 year. In this subgroup of elderly patients, the incidence rate of TVF was numerically lower compared to low-to-moderate-intensity statin group, which was consistent throughout extended study period of 24 months. 4) No data was available regarding side effects of statin and prescription rates of ezetimibe or PCSK9 inhibitor. Especially, PCSK9 inhibitor was not widely used in the study period due to governmental insurance coverage. 5) The study enrolled only South Korean population. Whether the results of the current study can be extrapolated to other ethnicities is unknown. Caution is needed in extrapolating these results outside of South Korea.

Nevertheless, we would like to emphasize that this study is the first research to answer the clinical impact of statin-intensity in elderly patients using large AMI registry.

## Conclusion

In this AMI cohort underwent PCI, high-intensity statin showed better clinical outcomes at 1-year than low-to-moderate intensity statin in younger patients. Meanwhile, in elderly patients, Meanwhile, the incidence rates of adverse clinical events were not statistically different between high- and low-to-moderate-intensity statin in elderly patients. Further randomized study with large elderly population is warranted.

## Supporting information

S1 Data (XLSX)

S1 Appendix (DOCX)

## References

[1] Collet JP, Thiele H, Barbato E, Barthélémy O, Bauersachs J, Bhatt DL, et al. 2020 ESC Guidelines for the management of acute coronary syndromes in patients presenting without persistent ST-segment elevation. European heart journal. 2021;42(14):1289–367. Epub 2020/08/30. doi: 10.1093/eurheartj/ehaa575 . 32860058

[2] Amarenco P, Bogousslavsky J, Callahan A 3rd, Goldstein LB, Hennerici M, Rudolph AE, et al. High-dose atorvastatin after stroke or transient ischemic attack. The New England journal of medicine. 2006;355(6):549–59. Epub 2006/08/11. doi: 10.1056/NEJMoa061894 . 16899775

[3] Baigent C, Blackwell L, Emberson J, Holland LE, Reith C, Bhala N, et al. Efficacy and safety of more intensive lowering of LDL cholesterol: a meta-analysis of data from 170,000 participants in 26 randomised trials. Lancet (London, England). 2010;376(9753):1670–81. Epub 2010/11/12. doi: 10.1016/S0140-6736(10)61350-5 ; PubMed Central PMCID: PMC2988224. 21067804 PMC2988224

[4] de Lemos JA, Blazing MA, Wiviott SD, Lewis EF, Fox KA, White HD, et al. Early intensive vs a delayed conservative simvastatin strategy in patients with acute coronary syndromes: phase Z of the A to Z trial. Jama. 2004;292(11):1307–16. Epub 2004/09/01. doi: 10.1001/jama.292.11.1307 . 15337732

[5] Navarese EP, Kowalewski M, Andreotti F, van Wely M, Camaro C, Kolodziejczak M, et al. Meta-analysis of time-related benefits of statin therapy in patients with acute coronary syndrome undergoing percutaneous coronary intervention. The American journal of cardiology. 2014;113(10):1753–64. Epub 2014/05/06. doi: 10.1016/j.amjcard.2014.02.034 . 24792742

[6] Ray KK, Cannon CP, McCabe CH, Cairns R, Tonkin AM, Sacks FM, et al. Early and late benefits of high-dose atorvastatin in patients with acute coronary syndromes: results from the PROVE IT-TIMI 22 trial. Journal of the American College of Cardiology. 2005;46(8):1405–10. Epub 2005/10/18. doi: 10.1016/j.jacc.2005.03.077 . 16226162

[7] Schwartz GG, Olsson AG, Ezekowitz MD, Ganz P, Oliver MF, Waters D, et al. Effects of atorvastatin on early recurrent ischemic events in acute coronary syndromes: the MIRACL study: a randomized controlled trial. Jama. 2001;285(13):1711–8. Epub 2001/04/13. doi: 10.1001/jama.285.13.1711 . 11277825

[8] Oliveira JS, Pinheiro MB, Fairhall N, Walsh S, Chesterfield Franks T, Kwok W, et al. Evidence on Physical Activity and the Prevention of Frailty and Sarcopenia Among Older People: A Systematic Review to Inform the World Health Organization Physical Activity Guidelines. Journal of physical activity & health. 2020:1–12. Epub 2020/08/12. doi: 10.1123/jpah.2020-0323 . 32781432

[9] Prince MJ, Wu F, Guo Y, Gutierrez Robledo LM, O’Donnell M, Sullivan R, et al. The burden of disease in older people and implications for health policy and practice. Lancet (London, England). 2015;385(9967):549–62. Epub 2014/12/04. doi: 10.1016/S0140-6736(14)61347-7 . 25468153

[10] Jaguszewski M, Ghadri JR, Diekmann J, Bataiosu RD, Hellermann JP, Sarcon A, et al. Acute coronary syndromes in octogenarians referred for invasive evaluation: treatment profile and outcomes. Clinical research in cardiology: official journal of the German Cardiac Society. 2015;104(1):51–8. Epub 2014/08/22. doi: 10.1007/s00392-014-0756-5 . 25142902

[11] Mach F, Baigent C, Catapano AL, Koskinas KC, Casula M, Badimon L, et al. 2019 ESC/EAS Guidelines for the management of dyslipidaemias: lipid modification to reduce cardiovascular risk. European heart journal. 2020;41(1):111–88. Epub 2019/09/11. doi: 10.1093/eurheartj/ehz455 . 31504418

[12] Grundy SM, Stone NJ, Bailey AL, Beam C, Birtcher KK, Blumenthal RS, et al. 2018 AHA/ACC/AACVPR/AAPA/ABC/ACPM/ADA/AGS/APhA/ASPC/NLA/PCNA Guideline on the Management of Blood Cholesterol: A Report of the American College of Cardiology/American Heart Association Task Force on Clinical Practice Guidelines. Circulation. 2019;139(25):e1082–e143. Epub 2018/12/28. doi: 10.1161/CIR.0000000000000625 ; PubMed Central PMCID: PMC7403606. 30586774 PMC7403606

[13] Jacobson TA, Maki KC, Orringer CE, Jones PH, Kris-Etherton P, Sikand G, et al. National Lipid Association Recommendations for Patient-Centered Management of Dyslipidemia: Part 2. Journal of clinical lipidology. 2015;9(6 Suppl):S1–122.e1. Epub 2015/12/25. doi: 10.1016/j.jacl.2015.09.002 . 26699442

[14] Koopman C, Vaartjes I, Heintjes EM, Spiering W, van Dis I, Herings RM, et al. Persisting gender differences and attenuating age differences in cardiovascular drug use for prevention and treatment of coronary heart disease, 1998–2010. European heart journal. 2013;34(41):3198–205. Epub 2013/09/21. doi: 10.1093/eurheartj/eht368 . 24046432

[15] Chou R, Dana T, Blazina I, Daeges M, Jeanne TL. Statins for Prevention of Cardiovascular Disease in Adults: Evidence Report and Systematic Review for the US Preventive Services Task Force. Jama. 2016;316(19):2008–24. Epub 2016/11/14. doi: 10.1001/jama.2015.15629 . 27838722

[16] Thygesen K, Alpert JS, Jaffe AS, Simoons ML, Chaitman BR, White HD, et al. Third universal definition of myocardial infarction. J Am Coll Cardiol. 2012;60(16):1581–98. Epub 2012/09/11. doi: 10.1016/j.jacc.2012.08.001 . 22958960

[17] Austin PC, Stuart EA. Moving towards best practice when using inverse probability of treatment weighting (IPTW) using the propensity score to estimate causal treatment effects in observational studies. Statistics in medicine. 2015;34(28):3661–79. Epub 2015/08/05. doi: 10.1002/sim.6607 ; PubMed Central PMCID: PMC4626409. 26238958 PMC4626409

[18] Rosenbaum PR. Model-based direct adjustment. J Am Stat Assoc. 1987;82:387–94.

[19] Therneau TM, Grambsch PM. Modeling survival data: extending the Cox model. New York: Springer. 2000.

[20] Rodriguez F, Maron DJ, Knowles JW, Virani SS, Lin S, Heidenreich PA. Association of Statin Adherence With Mortality in Patients With Atherosclerotic Cardiovascular Disease. JAMA cardiology. 2019;4(3):206–13. Epub 2019/02/14. doi: 10.1001/jamacardio.2018.4936 ; PubMed Central PMCID: PMC6439552. 30758506 PMC6439552

[21] Chen PS, Lin SH, Lee CH, Lin HW, Li YH. Efficacy and Safety of High-intensity Statins in Patients With Acute Myocardial Infarction: An Asian Perspective. The Canadian journal of cardiology. 2020;36(6):886–92. Epub 2020/03/25. doi: 10.1016/j.cjca.2019.10.027 . 32204951

[22] Rosenson RS, Kent ST, Brown TM, Farkouh ME, Levitan EB, Yun H, et al. Underutilization of high-intensity statin therapy after hospitalization for coronary heart disease. J Am Coll Cardiol. 2015;65(3):270–7. Epub 2015/01/24. doi: 10.1016/j.jacc.2014.09.088 . 25614424

[23] Nanna MG, Navar AM, Wang TY, Mi X, Virani SS, Louie MJ, et al. Statin Use and Adverse Effects Among Adults >75 Years of Age: Insights From the Patient and Provider Assessment of Lipid Management (PALM) Registry. Journal of the American Heart Association. 2018;7(10). Epub 2018/05/10. doi: 10.1161/jaha.118.008546 ; PubMed Central PMCID: PMC6015311. 29739801 PMC6015311

[24] Cannon CP, Braunwald E, McCabe CH, Rader DJ, Rouleau JL, Belder R, et al. Intensive versus moderate lipid lowering with statins after acute coronary syndromes. The New England journal of medicine. 2004;350(15):1495–504. Epub 2004/03/10. doi: 10.1056/NEJMoa040583 . 15007110

[25] Tikkanen MJ, Holme I, Cater NB, Szarek M, Faergeman O, Kastelein JJ, et al. Comparison of efficacy and safety of atorvastatin (80 mg) to simvastatin (20 to 40 mg) in patients aged <65 versus >or = 65 years with coronary heart disease (from the Incremental DEcrease through Aggressive Lipid Lowering [IDEAL] study). The American journal of cardiology. 2009;103(5):577–82. Epub 2009/02/24. doi: 10.1016/j.amjcard.2008.10.029 . 19231315

[26] Kwak A, Kim JH, Choi CU, Kim IW, Oh JM, Kim K. Comparative effectiveness of statins in secondary prevention among the older people aged 75 years and over. International journal of clinical pharmacy. 2019;41(2):460–9. Epub 2019/03/14. doi: 10.1007/s11096-019-00810-w . 30864086

[27] Foody JM, Rathore SS, Galusha D, Masoudi FA, Havranek EP, Radford MJ, et al. Hydroxymethylglutaryl-CoA reductase inhibitors in older persons with acute myocardial infarction: evidence for an age-statin interaction. Journal of the American Geriatrics Society. 2006;54(3):421–30. Epub 2006/03/23. doi: 10.1111/j.1532-5415.2005.00635.x ; PubMed Central PMCID: PMC2797316. 16551308 PMC2797316

[28] Rodriguez F, Maron DJ, Knowles JW, Virani SS, Lin S, Heidenreich PA. Association Between Intensity of Statin Therapy and Mortality in Patients With Atherosclerotic Cardiovascular Disease. JAMA cardiology. 2017;2(1):47–54. Epub 2016/11/10. doi: 10.1001/jamacardio.2016.4052 . 27829091

[29] Efficacy and safety of statin therapy in older people: a meta-analysis of individual participant data from 28 randomised controlled trials. Lancet (London, England). 2019;393(10170):407–15. Epub 2019/02/05. doi: 10.1016/S0140-6736(18)31942-1 ; PubMed Central PMCID: PMC6429627. 30712900 PMC6429627

[30] Strandberg TE, Tilvis RS, Pitkala KH, Miettinen TA. Cholesterol and glucose metabolism and recurrent cardiovascular events among the elderly: a prospective study. J Am Coll Cardiol. 2006;48(4):708–14. Epub 2006/08/15. doi: 10.1016/j.jacc.2006.04.081 . 16904538

[31] Bach RG, Cannon CP, Giugliano RP, White JA, Lokhnygina Y, Bohula EA, et al. Effect of Simvastatin-Ezetimibe Compared With Simvastatin Monotherapy After Acute Coronary Syndrome Among Patients 75 Years or Older: A Secondary Analysis of a Randomized Clinical Trial. JAMA cardiology. 2019;4(9):846–54. Epub 2019/07/18. doi: 10.1001/jamacardio.2019.2306 ; PubMed Central PMCID: PMC6647004. 31314050 PMC6647004

