# Supplementary material for: Clinical impact of statin intensity according to age in patients with acute myocardial infarction
Source: PLoS One. 2022 Jun 15;17(6):e0269301. doi: 10.1371/journal.pone.0269301 (PMC9200343; doi:10.1371/journal.pone.0269301)

**Supplemental Material**

**Supplementary Appendix**

This appendix has been provided by the authors to give readers additional information about their work. Supplement to: K Lee, M Lee, DW Kim, et al. “Clinical Impact of Statin Intensity According to Age in Patients with Acute Myocardial Infarction”.

**Appendix Table 1. Event rates and hazard ratios for clinical endpoints at 1 year among overall population.**

**Appendix Table 2. Demographics after propensity-score matching.**

**Appendix Table 3. Event rates and hazard ratios for clinical outcomes in maintenance phase after propensity-score matching from 1 month to 12 months.**

**Appendix Table 4. Univariate and multivariate predictors of target vessel failure from 1 month to 12 months.**

**Appendix Table 5. Subgroup analysis of event rates and hazard ratios for target-vessel failure among patients with continuing statin-intensity at 1 year.**

**Appendix Figure 1. Monthly incidence rates for clinical outcomes.**

† p value by Cochran-Armitage trend testing between acute phase (<1 month) and maintenance phase (from 1 month to 12 months)

*incidence rate of clinical outcome at 1 month

**mean value of incidence rate of clinical outcome from 1 month to 12 months

TVF, target vessel failure; CV, cardiovascular; TV-MI, target-vessel myocardial infarction; TLR, target lesion revascularization.

**Appendix Figure 2. Adjusted** **Kaplan-Meier curves for the secondary end points from 1 month to 12 months using inverse probability weighting.**

A-B) Cardiovascular death; C-D) Target vessel myocardial infarction; E-F) Target lesion revascularization.

Left panels (A,C,E) represented clinical outcomes in younger patients (<75 years old) and right panels (B,D,F) in elderly patients (≥75 years old).

CV, cardiovascular; TV-MI, target vessel myocardial infarction; TLR, target lesion revascularization.

**Appendix Figure 3. Kaplan-Meier curves for the clinical outcomes after propensity-score matching from 1 month to 12 months.**

**Appendix Table 1.**

| **Outcomes** | **Event rates at 1 year (n/%)*** | | **Crude** | | **Multivariate Adjusted**† | |
| --- | --- | --- | --- | --- | --- | --- |
|  | **Less intensive (n=6220)** | **High-intensity (n=1876)** | **HR** (95% CI)** | **P** | **HR (95% CI)** | **P** |
| TVF | 512 (8.2) | 142 (7.6) | 0.94 (0.78-1.13) | 0.48 | 0.9 (0.74-1.09) | 0.28 |
| All-cause death | 248 (4.0) | 88 (4.7) | 1.2 (0.94-1.53) | 0.14 | 1.12 (0.87-1.45) | 0.38 |
| Cardiovascular death | 191 (3.1) | 69 (3.7) | 1.22 (0.93-1.61) | 0.15 | 1.08 (0.81-1.46) | 0.59 |
| TV-MI | 48 (0.8) | 16 (0.9) | 1.13 (0.64-1.98) | 0.68 | 1.08 (0.6-1.93) | 0.81 |
| TLR | 318 (5.1) | 69 (3.7) | 0.73 (0.56-0.95) | 0.019 | 0.74 (0.57-0.96) | 0.025 |

*Event rates were derived from the Kaplan-Meier estimates.
** Hazard ratio is the risk of high-intensity statin for clinical outcomes compared with that of less intensive statin.
†adjusted by covariates including diabetes mellitus, chronic renal disease, peripheral artery disease, atrial fibrillation, left ventricle ejection fraction, left main disease, use of intravascular image, and total stent number.
HR, hazard ratio; CI, confidence interval; TVF, target vessel failure; TV-MI, target-vessel myocardial infarction; TLR, target lesion revascularization.

**Appendix Table 2.**

|  | **<75 years old** | | | | **≥75 years old** | | | |
| --- | --- | --- | --- | --- | --- | --- | --- | --- |
| **Characteristics** | **Low-to-moderate intensity (n = 1398)** | **High-intensity (n = 1398)** | **P** | **SMD** | **Low-to-moderate intensity (n = 305)** | **High-intensity (n = 305)** | **P** | **SMD** |
| **Baseline patients characteristics** | | | | | | | | |
| Age (years) | 57.3 ± 10.4 | 57.8 ± 10.0 | 0.24 | 0.045 | 79.9 ± 5.1 | 80.0 ± 4.8 | 0.77 | 0.024 |
| Male | 1153 (82.5) | 1144 (81.8) | 0.69 | 0.017 | 157 (51.5) | 157 (51.5) | 1.00 | <0.001 |
| Hypertension | 592 (42.3) | 654 (46.8) | 0.02 | 0.089 | 198 (64.9) | 199 (65.2) | 1.00 | 0.007 |
| Diabetes mellitus | 372 (26.6) | 377 (27.0) | 0.86 | 0.008 | 100 (32.8) | 98 (32.1) | 0.93 | 0.014 |
| Hyperlipidemia | 241 (17.2) | 309 (22.1) | 0.001 | 0.12 | 43 (14.1) | 41 (13.4) | 0.91 | 0.019 |
| Current smoker | 704 (50.4) | 697 (49.9) | 0.82 | 0.010 | 43 (14.1) | 50 (16.4) | 0.50 | 0.064 |
| Clinical diagnosis |  |  | 0.04 | 0.079 |  |  | 0.26 | 0.099 |
| STEMI | 805 (57.6) | 750 (53.6) |  |  | 149 (48.9) | 134 (43.9) |  |  |
| NSTEMI | 593 (42.4) | 648 (46.4) |  |  | 156 (51.1) | 171 (56.1) |  |  |
| CKD | 18 (1.3) | 26 (1.9) | 0.29 | 0.046 | 4 (1.3) | 6 (2.0) | 0.75 | 0.052 |
| Prior MI | 19 (1.4) | 19 (1.4) | 1.00 | <0.001 | 8 (2.6) | 9 (3.0) | 1.00 | 0.020 |
| Prior PCI | 53 (3.8) | 56 (4.0) | 0.84 | 0.011 | 25 (8.2) | 18 (5.9) | 0.34 | 0.090 |
| Prior CABG | 1 (0.1) | 1 (0.1) | 1.00 | <0.001 | 1 (0.3) | 2 (0.7) | 1.00 | 0.047 |
| Prior CVA | 63 (4.5) | 68 (4.9) | 0.72 | 0.017 | 33 (10.8) | 33 (10.8) | 1.00 | <0.001 |
| PAD | 7 (0.5) | 5 (0.4) | 0.77 | 0.022 | 1 (0.3) | 1 (0.3) | 1.00 | <0.001 |
| AF | 32 (2.3) | 14 (1.0) | 0.011 | 0.101 | 13 (4.3) | 7 (2.3) | 0.26 | 0.111 |
| LV EF, % | 54.6 ± 10.3 | 54.5 ± 10.5 | 0.76 | 0.012 | 51.1 ± 12.1 | 51.1 ± 11.7 | 0.97 | 0.003 |
| Total Cholesterol | 183 (159-214) | 184 (157-213) | 0.79 | 0.010 | 168 (141-195) | 169 (140-199) | 0.55 | 0.095 |
| Triglyceride | 106 (71-160) | 123 (81-174) | <0.001 | 0.145 | 80 (57-115) | 89 (63-126) | 0.019 | 0.082 |
| HDL Cholesterol | 40 (34-47) | 39 (34-46) | 0.09 | 0.055 | 41 (35-49) | 40 (34-48) | 0.23 | 0.122 |
| LDL Cholesterol | 119 (96-144) | 120 (96-145) | 0.58 | 0.031 | 107 (81-129) | 107 (81-132) | 0.57 | 0.073 |
| **Discharge medication** |  |  |  |  |  |  |  |  |
| Aspirin | 1383 (98.9) | 1375 (98.5) | 0.40 | 0.038 | 299 (98.0) | 303 (99.3) | 0.29 | 0.115 |
| P2Y12 inhibitor | 1369 (98.3) | 1378 (98.8) | 0.34 | 0.042 | 299 (98.4) | 303 (99.3) | 0.29 | 0.093 |
| **Lesion and Procedural characteristics** | | | | | | | | |
| Radial access | 297 (21.2) | 295 (21.1) | 0.96 | 0.004 | 76 (24.9) | 53 (17.4) | 0.029 | 0.201 |
| LM involved | 91 (6.5) | 90 (6.4) | 1.00 | 0.003 | 18 (5.9) | 17 (5.6) | 1.00 | 0.014 |
| pLAD involved | 609 (43.6) | 616 (44.1) | 0.82 | 0.010 | 141 (46.2) | 132 (43.3) | 0.52 | 0.059 |
| Disease extent |  |  | 0.67 | 0.047 |  |  | 0.72 | 0.117 |
| 1VD | 694 (49.6) | 671 (48.0) |  |  | 123 (40.4) | 125 (41.0) |  |  |
| 2VD | 439 (31.4) | 467 (33.4) |  |  | 95 (31.1) | 94 (30.8) |  |  |
| 3VD | 265 (19.0) | 260 (18.6) |  |  | 87 (28.5) | 86 (28.2) |  |  |
| Complex PCI | 587 (42.0) | 603 (43.1) | 0.57 | 0.023 | 130 (42.6) | 136 (44.6) | 0.68 | 0.040 |
| Total stent number | 1.61 ± 0.90 | 1.64 ± 0.90 | 0.37 | 0.034 | 1.68 ± 0.85 | 1.62 ± 0.88 | 0.40 | 0.068 |
| Mean stent diameter | 3.22 ± 0.43 | 3.19 ± 0.52 | 0.09 | 0.064 | 3.05 ± 0.35 | 3.08 ± 0.76 | 0.64 | 0.038 |
| Total stent length | 34.56 ± 21.8 | 34.89 ± 22.6 | 0.70 | 0.015 | 34.92 ± 20.1 | 34.18 ± 20.8 | 0.66 | 0.036 |
| IVUS use | 349 (24.7) | 291 (20.8) | 0.015 | 0.094 | 44 (14.4) | 52 (17.0) | 0.44 | 0.072 |

SMD, standardised mean difference; STEMI, ST-segment elevation myocardial infarction; NSTEMI, non ST-segment elevation myocardial infarction; CKD, chronic kidney disease; MI, myocardial infarction; PCI, percutaneous coronary intervention; CABG, coronary artery bypass grafting surgery; CVA, cerebrovascular attack; PAD, peripheral artery disease; AF, atrial fibrillation; LV EF, left ventricle ejection fraction; HDL, high-density lipoprotein; LDL, low-density lipoprotein; LM, left main; pLAD, proximal left anterior descending artery; VD, vessel disease; IVUS, intravascular ultrasound.

**Appendix Table 3.**

| **Outcomes** | **Event Rates at 1-12 Month (n/%*)** | | **Crude** | | **Multivariate Adjusted**† | |  |
| --- | --- | --- | --- | --- | --- | --- | --- |
|  | **Less intensive** | **High-intensity** | **HR (95% CI)** | **P** | **HR (95% CI)** | **P** | **P_interaction_** |
| **Overall** |  |  |  |  |  |  |  |
| TVF | 130 (8.0) | 106 (6.6) | 0.83 (0.64-1.07) | 0.15 | 0.80 (0.62-1.04) | 0.10 | 0.10 |
| All-cause death | 44 (2.7) | 60 (3.7) | 1.4 (0.95-2.07) | 0.09 | 1.41 (0.93-2.12) | 0.11 | 0.63 |
| CV death | 34 (2.1) | 45 (2.8) | 1.36 (0.87-2.13) | 0.18 | 1.3 (0.81-2.08) | 0.28 | 0.41 |
| TV-MI | 13 (0.8) | 8 (0.5) | 0.63 (0.26-1.53) | 0.31 | 0.55 (0.21-1.39) | 0.20 | >0.99 |
| TLR | 92 (5.7)** | 60 (3.8)** | 0.66 (0.48-0.92) | 0.013 | 0.65 (0.46-0.91) | 0.01 | 0.80 |
| **< 75 years old** |  |  |  |  |  |  |  |
| TVF | 329 (6.9)** | 79 (5.5)** | 0.74 (0.55-0.99) | 0.046 | 0.7 (0.51-0.95) | 0.023 |  |
| All-cause death | 23 (1.7) | 28 (2.1) | 1.27 (0.73-2.2) | 0.4 | 1.28 (0.7-2.34) | 0.42 |  |
| CV death | 17 (1.2) | 19 (1.4) | 1.16 (0.6-2.24) | 0.65 | 1.08 (0.52-2.24) | 0.83 |  |
| TV-MI | 13 (1.0) | 8 (0.6) | 0.64 (0.27-1.54) | 0.32 | 0.53 (0.21-1.35) | 0.19 |  |
| TLR | 81 (6.0)** | 52 (4.0)** | 0.66 (0.47-0.94) | 0.02 | 0.64 (0.45-0.91) | 0.01 |  |
| **≥ 75 years old** |  |  |  |  |  |  |  |
| TVF | 131 (10.8) | 38 (11.7) | 1.12 (0.68-1.86) | 0.65 | 1.1 (0.66-1.85) | 0.72 |  |
| All-cause death | 113 (9.2) | 39 (11.8) | 1.48 (0.85-2.56) | 0.17 | 1.47 (0.84-2.6) | 0.18 |  |
| CV death | 17 (6.3) | 26 (9.0) | 1.48 (0.8-2.73) | 0.21 | 1.45 (0.77-2.73) | 0.25 |  |
| TV-MI | 0 | 0 | NA | NA | NA | NA |  |
| TLR | 11 (4.3) | 8 (3.0) | 0.7 (0.28-1.73) | 0.44 | 0.71 (0.28-1.82) | 0.48 |  |

*Event rates were derived from the Kaplan-Meier estimates.
**Hazard ratio is the risk of high-intensity statin for clinical outcomes compared with that of less intensive statin.
†adjusted by covariates including diabetes mellitus, chronic renal disease, peripheral artery disease, atrial fibrillation, left ventricle ejection fraction, left main disease, and total stent number.
HR, hazard ratio; CI, confidence interval; TVF, target vessel failure; CV, cardiovascular; TV-MI, target-vessel myocardial infarction; TLR, target lesion revascularization.

**Appendix Table 4.**

| **Variables** | **Univariate** | | **Multivariate** | |
| --- | --- | --- | --- | --- |
|  | **HR (95% CI)** | **P** | **HR (95% CI)** | **P** |
| **Overall** |  |  |  |  |
| High-intensity statin* | 0.84 (0.68-1.03) | 0.09 | 0.86 (0.7-1.06) | 0.16 |
| Age | 1.02 (1.01-1.03) | <0.001 | 1.02 (1.01-1.02) | <0.001 |
| Diabetes mellitus | 1.43 (1.2-1.69) | <0.001 | 1.22 (1.02-1.46) | 0.032 |
| Chronic kidney disease† | 3.2 (2.11-4.86) | <0.001 | 2.62 (1.7-4.04) | <0.001 |
| PAD | 2.91 (1.38-6.14) | 0.005 | 2.44 (1.16-5.17) | 0.019 |
| AF | 1.85 (1.23-2.78) | 0.003 | 1.58 (1.05-2.39) | 0.029 |
| LV EF, % | 0.98 (0.97-0.99) | <0.001 | 0.99 (0.98-0.99) | <0.001 |
| LM involved | 1.67 (1.26-2.21) | <0.001 | 1.5 (1.12-2.0) | 0.007 |
| Total stent number | 1.21 (1.11-1.31) | <0.001 | 1.18 (1.06-1.32) | 0.002 |
| IVUS use | 0.8 (0.65-1.0) | 0.05 | 0.78 (0.62-0.98) | 0.032 |
| **< 75 years old** |  |  |  |  |
| High-intensity statin* | 0.77 (0.59-0.99) | 0.04 | 0.77 (0.59-0.99) | 0.04 |
| Diabetes mellitus | 1.44 (1.17-1.77) | <0.001 | 1.25 (1.01-1.55) | 0.039 |
| Chronic kidney disease† | 3.05 (1.85-5.03) | <0.001 | 2.35 (1.41-3.93) | 0.001 |
| PAD | 3.68 (1.52-8.89) | 0.004 | 3.28 (1.36-7.95) | 0.008 |
| LV EF, % | 0.98 (0.98-0.99) | <0.001 | 0.99 (0.98-1.0) | 0.019 |
| LM involved | 1.81 (1.29-2.54) | <0.001 | 1.64 (1.15-2.34) | 0.006 |
| Total stent number | 1.24 (1.13-1.37) | <0.001 | 1.2 (1.08-1.33) | <0.001 |
| **≥ 75 years old** |  |  |  |  |
| High-intensity statin* | 1.07 (0.74-1.56) | 0.70 | 1.1 (0.76-1.59) | 0.63 |
| Chronic kidney disease† | 3.94 (1.85-8.4) | <0.001 | 3.44 (1.59-7.43) | 0.002 |
| LV EF, % | 0.98 (0.97-0.99) | 0.006 | 0.98 (0.97-0.99) | 0.007 |
| IVUS use | 0.61 (0.37-0.99) | 0.047 | 0.61 (0.37-1.0) | 0.049 |

*Hazard ratio is the risk of high-intensity statin for clinical outcomes compared with that of less intensive statin.
†Chronic kidney disease was defined as eGFR < 60 mL/min/1.73m^2^.
‡Not retained as an independent predictor in the multivariate analysis.
HR, hazard ratio; CI, confidence interval; CVA, cerebrovascular attack; PAD, peripheral vascular disease; AF, atrial fibrillation; LV EF, left ventricle ejection fraction; LM, left main; PCI, percutaneous coronary intervention; IVUS, intravascular ultrasound.

**Appendix Table 5.**

| **TVF** | **Event Rates (n/%*)** | | **Multivariate adjusted**† | |
| --- | --- | --- | --- | --- |
|  | **Less intensive** | **High-intensity** | **HR** (95% CI)** | **P** |
| **At 1-12 months** |  |  |  |  |
| **Overall** | 285 (5.9) | 39 (3.8) | 0.64 (0.46-0.9) | 0.01 |
| **< 75 years old** | 238 (6.0) | 34 (3.8) | 0.63 (0.44-0.9) | 0.012 |
| **≥ 75 years old** | 47 (5.2) | 5 (3.8) | 0.7 (0.28-1.77) | 0.46 |
| **At 1-24 months** |  |  |  |  |
| **Overall** | 478 (9.8) | 76 (7.4) | 0.75 (0.59-0.96) | 0.02 |
| **< 75 years old** | 384 (9.7) | 66 (7.4) | 0.76 (0.59-0.99) | 0.044 |
| **≥ 75 years old** | 94 (10.5) | 10 (7.5) | 0.71 (0.37-1.37) | 0.305 |

†adjusted by covariates including diabetes mellitus, chronic renal disease, peripheral artery disease, atrial fibrillation, left ventricle ejection fraction, left main disease, and total stent number.
*Event rates were derived from the Kaplan-Meier estimates. Hazard ratio is the risk of high-intensity statin for clinical outcomes compared with that of less intensive statin.
**P value by log-rank test was less than 0.05.
HR, hazard ratio; CI, confidence interval; TVF, target vessel failure.

**Appendix Figure 1.**

**

**

**
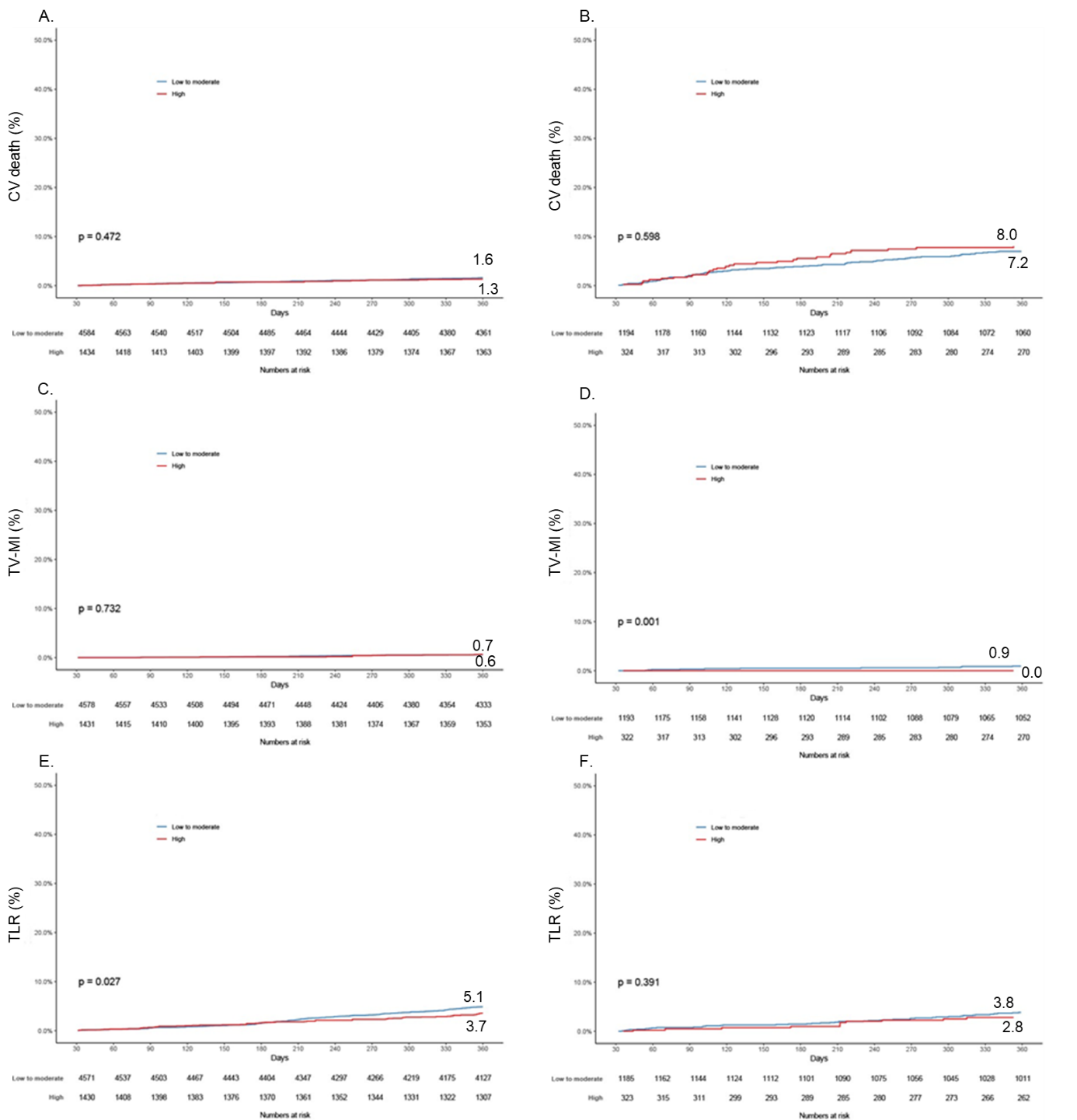
Appendix Figure 2.**

**Appendix Figure 3.**


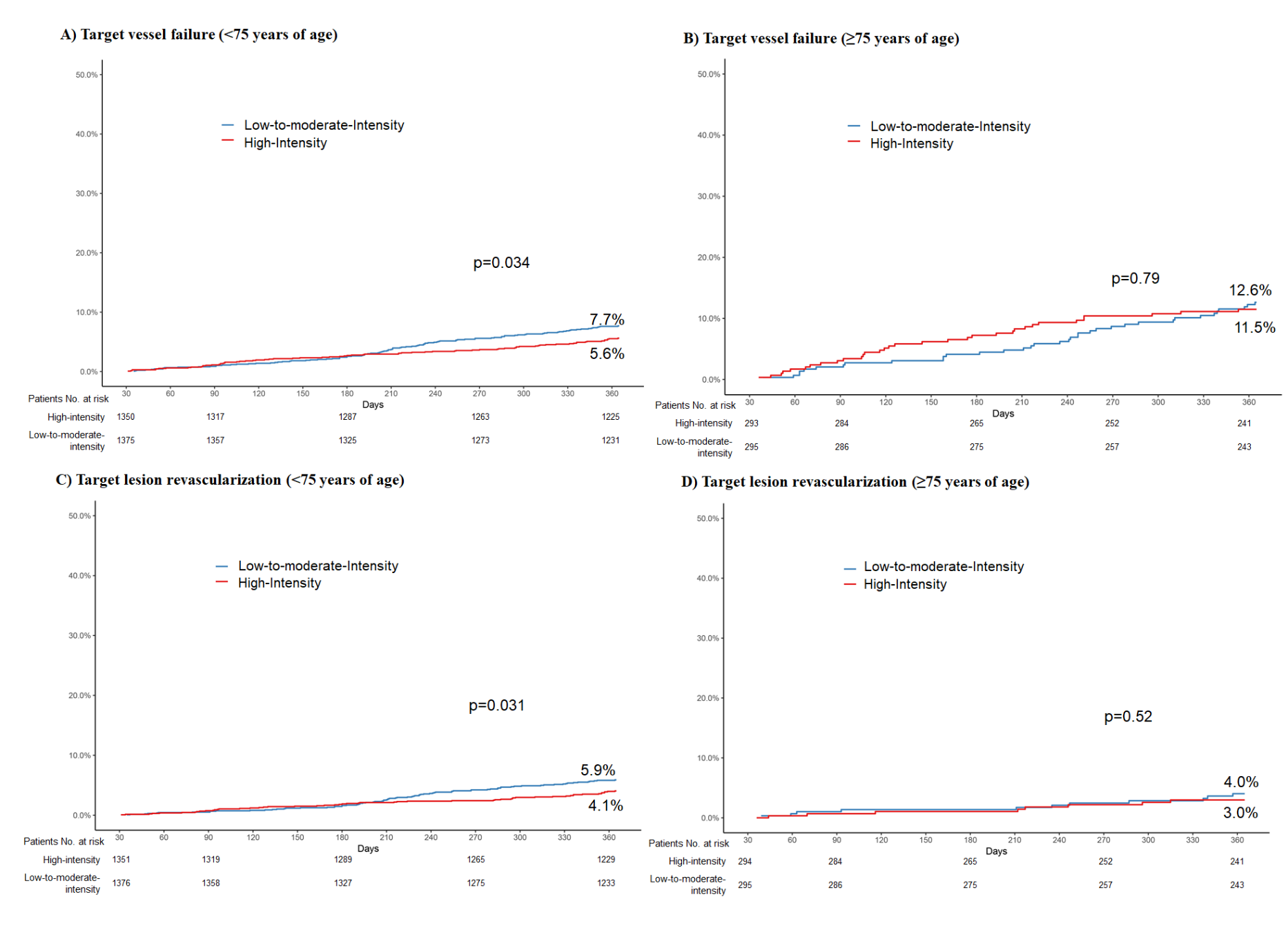

Supplement: S1 Appendix — (DOCX) [file pone.0269301.s002.docx]
