# Supplementary material for: Clinical impact of statin intensity according to age in patients with acute myocardial infarction
Source: PLoS One. 2022 Jun 15;17(6):e0269301. doi: 10.1371/journal.pone.0269301 (PMC9200343; doi:10.1371/journal.pone.0269301)
Supplement: Attachment — Submitted filename: Response to Reviewers-FINAL.docx [file pone.0269301.s003.docx]

**Revision Response Letter**

Ref. No: PONE-D-21-14585

Title: Clinical Impact of Statin Intensity According to Age in Patients with Acute Myocardial Infarction.

Dear Dr. Emily Chenette

Academic Editor of PLOS ONE

We thank you, the reviewers and editorial staff for the constructive and valuable comments regarding our manuscript. We now provide a revised manuscript for consideration of publication in PLOS ONE. We have carefully considered each comment and attached a point-by-point-response to the reviewers’ comments. We hope that the paper will be of substantial interest to the readership of PLOS ONE. All authors concur with the submission and the material has not been published previously or is not under consideration for publication elsewhere.

On behalf of the authors and with best regards

**Mahn-Won Park, M.D., Ph.D., Associate Professor**

Department of Cardiology, Daejeon St. Mary’s Hospital, College of Medicine,

The Catholic University of Korea

64, Daeheung-ro, Jung-gu, Daejeon, 34943, Korea

Phone: +82-10-3800-2817, E-mail: pmw6193@catholic.ac.kr

We thank to the editor and reviewers for their time and input, and their precious comments.

**1. Please ensure that your manuscript meets PLOS ONE's style requirements, including those for file naming.**

**Answer :** We changed the title and subtitle with capitalization only the first word.

Modified text : Line 3

**Clinical impact of statin intensity according to age in patients with
acute myocardial infarction**

*Short title*: Statin intensity in elderly patients

**Answer :** We changed the author names without titles (MD, PhD).

Modified text : Line 8

Kyusup Lee^1,2^, Myunhee Lee^1,2^, Dae-Won Kim^1,2^, Jinseob Kim^3^, Sungmin Lim^1,4^,
Eun Ho Choo^1,9^, Chan Joon Kim^1,4^, Chul Soo Park^1,5^, Hee Yeol Kim^1,6^, Ki-Dong Yoo^1,7^,
Doo Soo Jeon^1,8^, Kiyuk Chang^1,9^, Ho Joong Youn^1,9^, Wook-Sung Chung^1,9^, Min Chul Kim^10^,
Myung Ho Jeong^10^, Youngkeun Ahn^10^, Jongbum Kwon^11^, and Mahn-Won Park^1,2^.

**2. In your Data Availability statement, you have not specified where the minimal data set underlying the results described in your manuscript can be found. PLOS defines a study's minimal data set as the underlying data used to reach the conclusions drawn in the manuscript and any additional data required to replicate the reported study findings in their entirety. All PLOS journals require that the minimal data set be made fully available. For more information about our data policy, please see http://journals.plos.org/plosone/s/data-availability.**

**Answer :** We added the ‘Data Avilability statement’ in the cover letter.

**Data Availability:** The data cannot be made public due to the protection of the patients’ personal information. Data are available from the Department of Cardiology in the Daejeon St. Mary's Hospital (contact via pmw6193@catholic.ac.kr - the person in charge of data management at the Department of Cardiology in the Daejeon St. Mary's Hospital) for researchers who meet the criteria for access to confidential data.

**3. PLOS requires an ORCID iD for the corresponding author in Editorial Manager on papers submitted after December 6th, 2016. Please ensure that you have an ORCID iD and that it is validated in Editorial Manager. To do this, go to ‘Update my Information’ (in the upper left-hand corner of the main menu), and click on the Fetch/Validate link next to the ORCID field. This will take you to the ORCID site and allow you to create a new iD or authenticate a pre-existing iD in Editorial Manager. Please see the following video for instructions on linking an ORCID iD to your Editorial Manager account:** [**https://www.youtube.com/watch?v=_xcclfuvtxQ**](https://www.youtube.com/watch?v=_xcclfuvtxQ)

**Answer:** Mahn-Won Park, ORCID Id: 0000-0001-5293-8461. Thanks.

**4. Please include captions for your Supporting Information files at the end of your manuscript, and update any in-text citations to match accordingly. Please see our Supporting Information guidelines for more information:** [**http://journals.plos.org/plosone/s/supporting-information**](http://journals.plos.org/plosone/s/supporting-information)

**Reviewers' comments**

**Reviewer #1:** The manuscript by Lee et al examines more than 10,000 patients with AMI and the clinical impact of statin use. Specifically, they demonstrate better clinical outcomes at 1-year in patients <75 treated with a high-intensity statin compared with a low-to-moderate-intensity statin. There are some concerns that should be addressed by the authors.

**1. A major emphasis of the manuscript is that "less-intensive statin [sic] showed feasible and acceptable clinical outcomes compared to high-intensity statin in elderly patients." Yet, the authors themselves acknowledge that the findings are subject to selection bias from the physicians making treatment decisions and the limited sample size of older adult patients included in the propensity matched cohort (just 308 older adults matched) that prevents the comparison between statin-intensity groups in elderly patients. Thus, I don't think the authors can make any firm conclusions from that particular comparison and the current wording of both the abstract and conclusion is a bit misleading.**

**Answer :** Thanks for your valuable comment. As you pointed out, our findings are subject to selection bias and confounding factors because of its non-randomized, observational design. This is inherent limitaion of real-world observational studies. Thus, to minimize these biases, we performed rigorous adjustments using a multivariable Cox proportional hazard regression model and PS matching, but hidden bias may still remain because of the influence of unmeasured confounding factors. We already mentioned this in the ‘Limitation’ section in the main manuscript. Therefore, because these factors may make it difficult to draw definite conclusions on the current issues, our findings should be considered with caution until additional large clinical studies with long-term follow-ups replicate our findings. However, it is also known that randomized controlled trial may not reflect real world clinical situation due to its strict inclusion criteria. In this regard, we would like to emphasize the strength of our study. First, our study is the first and the largest study investigating the clinical impact of statin intenstiy according to age in homogenous AMI population, even though the sample size of elderly patients is relatively small. Second, we only included AMI patients underwent PCI with DES. Currently, PCI with DES is regarded as the standard of treatment in AMI patients. Thus, we think that our findings can relflect current clinical practice in this population.

In addition, to respond your comments and to further support the results of our study, we additionally performed subgroup analysis with patients who were continuing the same intensity of statin. Because adherence and tolerance are important clinical aspects of medical treatment that affect clinical outcomes, we investigated the maintenance proportion of the same intensity of statin during 1 year after index AMI in total of 6,566 patients. In patients who received high-intensity statin at discharge, the proportion of patients who maintained the same intensity of statin was significantly lower in elderly patients than in younger patients (55.0% vs. 66.3%, p=0.0009), which was not shown in low-to-moderate-intensity statin group (99.1% vs. 98.1%, p=0.06) (please see below figure). In other words, de-escalation of statin-intensity was frequently ocurred in elderly patients treated with high-intensity statin.

In overall and younger patients, patients who maintained high-intensity statin showed significantly better clinical outcome in terms of TVF (overall: HR 0.64, 95% CI 0.46-0.9, p=0.01; younger patients: HR 0.63, 95% CI 0.44-0.9, p=0.012). However, in elderly patients, the efficacy for reducing adverse clinical outcomes did not differ between continuous use of high- and low-to-moderate-intensity statin (HR 0.7, 95% CI 0.28-1.77, p=0.46) (please see below table). These findings were consistent with the main result of our study. However, the incidence rate of TVF was numerically lower in high-intensity statin group compared to low-to-moderate-intensity statin group. To overcome the small number of event rates, we extended a study period to 24 months, which also showed a consistent result (Appendix Table 5).

Finally, to respond your comment, we tempered the conclusion as you recommended and added a text as below. We ask for your understanding. Thanks a lot.

**Figure. Age differences on maintenance proportion of same statin-intensity**

**Table. Subgroup analysis of event rates and hazard ratios for target-vessel failure among patients with continuing statin-intensity at 1 year.**

| **TVF** | **Event Rates (n/%*)** | | **Multivariate adjusted**† | |
| --- | --- | --- | --- | --- |
|  | **Less intensive** | **High-intensity** | **HR** (95% CI)** | **P** |
| **At 1-12 months** |  |  |  |  |
| **Overall** | 285 (5.9) | 39 (3.8) | 0.64 (0.46-0.9) | 0.01 |
| **< 75 years old** | 238 (6.0) | 34 (3.8) | 0.63 (0.44-0.9) | 0.012 |
| **≥ 75 years old** | 47 (5.2) | 5 (3.8) | 0.7 (0.28-1.77) | 0.46 |
| **At 1-24 months** |  |  |  |  |
| **Overall** | 478 (9.8) | 76 (7.4) | 0.75 (0.59-0.96) | 0.02 |
| **< 75 years old** | 384 (9.7) | 66 (7.4) | 0.76 (0.59-0.99) | 0.044 |
| **≥ 75 years old** | 94 (10.5) | 10 (7.5) | 0.71 (0.37-1.37) | 0.305 |

†adjusted by covariates including diabetes mellitus, chronic renal disease, peripheral artery disease, atrial fibrillation, left ventricle ejection fraction, left main disease, and total stent number.
*Event rates were derived from the Kaplan-Meier estimates. Hazard ratio is the risk of high-intensity statin for clinical outcomes compared with that of less intensive statin.
**P value by log-rank test was less than 0.05.
HR, hazard ratio; CI, confidence interval; TVF, target vessel failure.

**Original text :** Line 66, abstract

Meanwhile, in elderly patients, high-intensity statin showed similar clinical outcomes compared to low-to-moderate-intensity statin, which meant less-intensive statin showed feasible and acceptable clinical outcomes compared to high-intensity statin in elderly patients.

**Modified text :**

Meanwhile, in elderly patients, there is no difference in clinical outcomes according to statin intensity. Further randomized study with large population of elderly is warranted.

**Original text :** Line 342, conclusion

In elderly patients, less intensive statin showed feasible and acceptable clinical outcomes compared to high-intensity statin for secondary prevention considering compliance, concern about side effect and drug interaction.

**Modified text :**

Meanwhile, in elderly patients, clinical outcomes were not different between high-intenstiy and low-to-moderate-intensity statin. Further randomized study with large population of elderly is warranted.

**Original text :** Line 261, results

**Modified text :**

Subgroup analysis for clinical impact of statin-intensity among patients continuing statin-intensity

In order to further support the results of our study, we performed subgroup analysis with patients who were continuing the same intensity of statin. Because adherence and tolerance are important clinical aspects of medical treatment that affect clinical outcomes, we investigated the maintenance proportion of the same intensity of statin during 1 year after index AMI in total of 6,566 patients. In patients who received high-intensity statin at discharge, the proportion of patients who maintained the same intensity of statin was significantly lower in elderly patients than that of younger patients (55.0% vs. 66.3%, p=0.0009), which was not shown in low-to-moderate-intensity statin group (99.1% vs. 98.1%, p=0.06) (Figure 2). In other words, de-escalation of statin-intensity was frequently occurred in elderly patients treated with high-intensity statin.

In overall and younger patients, patients who maintained high-intensity statin showed significantly better clinical outcome in terms of TVF (overall: HR 0.64, 95% CI 0.46-0.9, p=0.01; younger patients: HR 0.63, 95% CI 0.44-0.9, p=0.012). However, in elderly patients, the efficacy for reducing adverse clinical outcomes did not differ between continuous use of high- and low-to-moderate-intensity statin (HR 0.7, 95% CI 0.28-1.77, p=0.46). These findings were consistent with the main results of our study. However, the incidence rate of TVF was numerically lower in high-intensity statin group compared to low-to-moderate-intensity statin group. To overcome the small number of event rates, we extended a study period to 24 months, which showed a consistent result (Appendix Table 5).

**Original text :** Line 328

Although propensity-score analyses were performed to adjust for potential selection bias, the unmeasured confounders might have affected the results.

**Modified text :**

However, to minimize the bias, we performed rigorous adjustments using a multivariable Cox proportional hazard regression model and PS matching and subgroup analysis with patients who were continuing the same statin-intensity during 1 year. Nevertheless, hidden bias may still remain because of the influence of unmeasured confounding factors. Therefore, our findings should be considered with caution until additional large clinical studies with long-term follow-ups replicate our findings.

**Figure 2. Age differences on maintenance proportion of statin-intensity.**

**Appendix Table 5. Subgroup analysis of event rates and hazard ratios for target-vessel failure among patients with continuing statin-intensity at 1 year.**

| **TVF** | **Event Rates (n/%*)** | | **Multivariate adjusted**† | |
| --- | --- | --- | --- | --- |
|  | **Less intensive** | **High-intensity** | **HR** (95% CI)** | **P** |
| **At 1-12 months** |  |  |  |  |
| **Overall** | 285 (5.9) | 39 (3.8) | 0.64 (0.46-0.9) | 0.01 |
| **< 75 years old** | 238 (6.0) | 34 (3.8) | 0.63 (0.44-0.9) | 0.012 |
| **≥ 75 years old** | 47 (5.2) | 5 (3.8) | 0.7 (0.28-1.77) | 0.46 |
| **At 1-24 months** |  |  |  |  |
| **Overall** | 478 (9.8) | 76 (7.4) | 0.75 (0.59-0.96) | 0.02 |
| **< 75 years old** | 384 (9.7) | 66 (7.4) | 0.76 (0.59-0.99) | 0.044 |
| **≥ 75 years old** | 94 (10.5) | 10 (7.5) | 0.71 (0.37-1.37) | 0.305 |

†adjusted by covariates including diabetes mellitus, chronic renal disease, peripheral artery disease, atrial fibrillation, left ventricle ejection fraction, left main disease, and total stent number.
*Event rates were derived from the Kaplan-Meier estimates. Hazard ratio is the risk of high-intensity statin for clinical outcomes compared with that of less intensive statin.
**P value by log-rank test was less than 0.05.
HR, hazard ratio; CI, confidence interval; TVF, target vessel failure.

**2. The finding that high-intensity statin use does not benefit older adults runs counter to larger prior analyses that support an association between high-intensity statin use and a survival advantage in older adults. This is more reason to temper the conclusions around statin intensity in older patients with MI.**
Rodriguez F, Maron DJ, Knowles JW, Virani SS, Lin S and Heidenreich PA. Association Between Intensity of Statin Therapy and Mortality in Patients With Atherosclerotic Cardiovascular Disease. JAMA Cardiol. 2017;2:47-54
CTT. Efficacy and safety of statin therapy in older people: a meta-analysis of individual participant data from 28 randomised controlled trials. Lancet. 2019;393:407-415.

**Answer :** We appreciate your valuable and attentive advice with references. With response to your comment No #1, We already tempered the conclusion as mentioned above and added those references that you suggested in the ‘Discussion’ session.

In general, elderly individuals have a shorter life expectancy and more comorbidities than younger people, so statins may have fewer benefits in this population. This is why in elderly people, the benefit and disadvantages of the treatment with statins should be put in balance, especially in those receiving high-intensity of statins. However, it is true that the amount of information is not enough regarding the effects of high-intensity statin therapy for elderly patients with ASCVD. For this reason, previous version of ACC/AHA cholesterol guideline recommended moderate-intensity statin therapy for patients older than 75 years of age. [1] The current guideline also recommends that in patients older than 75 years of age with clinical ASCVD, it is reasonable to initiate moderate- or high-intensity statin therapy after evaluation of the potential for ASCVD risk reduction, adverse effects, and drug–drug interactions, as well as patient frailty and patient preferences (COR IIa) [2]

There are several previous studies which showed the similar clinical efficacy of less-intensity statin compared to high-intensity statin in CAD population. [3,4] IDEAL (Incremental DEcrease through Aggressive Lipid Lowering) Study randomly assigned 8,888 patients age≤ 80 years with previous myocardial infarction to receive either intensive therapy with atorvastatin 80 mg/day or the standard statin therapy at the time (simvastatin 20 to 40 mg/day) to compare the ability of these regimens to lower cardiovascular risk. In the subgroup analysis, significant reductions in primary and secondary endpoints were observed only in patients <65 years of age compared to 65 to 80 years of age (please see below figure).[4]

**Figure. Intensive atorvastatin therapy reduced event rates in both younger and older patients.**

Especially, there has been various studies enrolling Asian population which have shown similar clinical efficacy of less-intensive statin compared to high-intensity statin. Our group reported that the efficacy of moderate-intensity statin therapy was comparable to that of high-intensity statin therapy in terms of improved clinical outcomes using national health insurance claims (moderate intensity group, n=23,863, high intensity group, n=9,073) data in South Korea after propensity matching analysis.[5]

In addition, a pharmacokinetic study also indicated that the greater effect of statins could be due in part to the difference in statin pharmacokinetics between East Asian and Western patients.[6] In prior studies with East Asian populations, lower-dose statin therapy showed similar therapeutic effects to those observed in Western populations using higher-dose statin therapy [7,8]. In addition, serial intravascular ultrasound studies with East Asian patients demonstrated that the regression of coronary atherosclerosis could be achieved by moderate-intensity statin therapy [8-10]. Thus, for generalizability of our results to other ethnicities, we added a text in the limitation section as below. As mentioned above, to respond your suggestion, we added two references that you mentioned in the ‘Discussion’ session. Even though those studies provided the clinical relevance of high-intensity statin in elderly population, they also have some limitations. Their study population was not homogenous AMI patients unlike our study. About 60% of study population were CAD not knowing information about revascularization in these studies.[11] Besides, with cautiously looking at the meta-analysis by Cholesterol Treatment Trialists’ Collaboration, 26 trials were analyzed statin therapy versus control, not comparison the intensity of statin.[12] Thus, we need well-powered large scale randomized studies regarding this topic.

Accordingly, although our findings may not be conclusive due to inherent limitations, we think that our results can provide an additional evidence for the statin intensity in elderly patients with AMI. Thanks a lot.

**Original text : Line 298, discussion**

Kwak A. et al.[19] reported that moderate-intensity statin can be more protective against ischemic cardiovascular and cerebrovascular events compared with high-intensity statin in patients aged 75 years and older. Foody J.M. et al.[20] showed that statin therapy was associated with a significant reduction in all-cause mortality in patients younger than 80, but not in patients aged 80 and older. Likewise, our findings are in line with the results of these previous studies.

**Modified text :**

IDEAL (Incremental DEcrease through Aggressive Lipid Lowering) Study randomly assigned 8,888 patients age ≤ 80 years with previous myocardial infarction to receive either intensive therapy with atorvastatin 80 mg/day or the standard statin therapy at the time (simvastatin 20 to 40 mg/day) to compare the ability of these regimens to lower cardiovascular risk. In the subgroup analysis, significant reductions in primary and secondary endpoints were observed only in patients <65 years of age compared to 65 to 80 years of age.[24] Kwak A. et al.[25] reported that moderate-intensity statin can be more protective against ischemic cardiovascular and cerebrovascular events compared with high-intensity statin in patients aged 75 years and older. Foody J.M. et al.[26] showed that statin therapy was associated with a significant reduction in all-cause mortality in patients younger than 80, but not in patients aged 80 and older. Although, some analyses reported an association between high-intensity statin use and a survival benefit in older adults recently,[27, 28] their study population was not homogenous AMI patients unlike our study. About 60% of study population were CAD not knowing information about revascularization in these studies.[27] Besides, with cautiously looking at the meta-analysis by Cholesterol Treatment Trialists’ Collaboration, 26 trials were analyzed statin therapy versus control, not comparison the intensity of statin.[28] Thus, we need well-powered large scale randomized studies regarding this topic.

**Original text : Line 334, discussion**

**Modified text :**

5) the study enrolled only South Korean population. Whether the results of the current study can be extrapolated to other ethnicities is unknown. Caution is needed in extrapolating these results outside of South Korea.

Reference

1. Stone NJ, Robinson J, Lichtenstein AH, Merz CNB, Blum CB, Eckel RH, et al. 2013 ACC/AHA guideline on the treatment of blood cholesterol to reduce atherosclerotic cardiovascular risk in adults: a report of the American College of Cardiology/American Heart Association Task Force on Practice Guidelines. Circulation. 2014;129(25 Suppl 2):S1–45.

2. Grundy SM, Stone NJ, Bailey AL, Beam C, Birtcher KK, Blumenthal RS, et al. 2018 AHA/ACC/AACVPR/AAPA/ABC/ACPM/ADA/AGS/APhA/ASPC/NLA/PCNA Guideline on the Management of Blood Cholesterol: A Report of the American College of Cardiology/American Heart Association Task Force on Clinical Practice Guidelines. Circulation. 2019;139(25):e1082-e143

3. Christopher P. Cannon, M.D., Eugene Braunwald, M.D., Carolyn H. et al. Intensive versus Moderate Lipid Lowering with Statins after Acute Coronary Syndromes. PROVE IT–TIMI 22 study. N Engl J Med 2004;350:1495-504.

4. Matti J Tikkanen 1, Ingar Holme, Nilo B Cater, Michael Szarek, Ole Faergeman et al. Comparison of efficacy and safety of atorvastatin (80 mg) to simvastatin (20 to 40 mg) in patients aged <65 versus >or=65 years with coronary heart disease (from the Incremental DEcrease through Aggressive Lipid Lowering [IDEAL] study). Am J Cardiol 2009;103:577-82

5. Park MW, Park GM, Han S, Yang Y, Kim YG, Roh JH, et al. Moderate-intensity versus high-intensity statin therapy in Korean patients with angina undergoing percutaneous coronary intervention with drug-eluting stents: A propensity-score matching analysis. PloS one. 2018;13(12):e0207889

6. Lee E, Ryan S, Birmingham B, Zalikowski J, March R, Ambrose H, et al. Rosuvastatin pharmacokinetics and pharmacogenetics in white and Asian subjects residing in the same environment. Clin Pharmacol Ther. 2005; 78: 330–341

7. Kong SH, Koo BK, Moon MK. Efficacy of Moderate Intensity Statins in the Treatment of Dyslipidemia in Korean Patients with Type 2 Diabetes Mellitus. Diabetes Metab J. 2017; 41: 23–30.

8. Lee CW, Kang SJ, Ahn JM, Song HG, Lee JY, Kim WJ, et al. Comparison of effects of atorvastatin (20mg) versus rosuvastatin (10 mg) therapy on mild coronary atherosclerotic plaques (from the ARTMAP trial). Am J Cardiol. 2012; 109: 1700–1704.

9. Hiro T, Kimura T, Morimoto T, Miyauchi K, Nakagawa Y, Yamagishi M, et al. Effect of intensive statin therapy on regression of coronary atherosclerosis in patients with acute coronary syndrome: a multicenter randomized trial evaluated by volumetric intravascular ultrasound using pitavastatin versus atorvastatin (JAPAN-ACS [Japan assessment of pitavastatin and atorvastatin in acute coronary syndrome] study). J Am Coll Cardiol. 2009; 54: 293–302.

10. Takayama T, Hiro T, Yamagishi M, Daida H, Hirayama A, Saito S, et al. Effect of rosuvastatin on coronary atheroma in stable coronary artery disease: multicenter coronary atherosclerosis study measuring effects of rosuvastatin using intravascular ultrasound in Japanese subjects (COSMOS). Circ J. 2009;73: 2110–2117.

11. Rodriguez F, Maron DJ, Knowles JW, Virani SS, Lin S, Heidenreich PA. Association Between Intensity of Statin Therapy and Mortality in Patients With Atherosclerotic Cardiovascular Disease. JAMA cardiology. 2017;2(1):47-54. Epub 2016/11/10. doi: 10.1001/jamacardio.2016.4052. PubMed PMID: 27829091.

12. Efficacy and safety of statin therapy in older people: a meta-analysis of individual participant data from 28 randomised controlled trials. Lancet (London, England). 2019;393(10170):407-15. Epub 2019/02/05. doi: 10.1016/s0140-6736(18)31942-1. PubMed PMID: 30712900; PubMed Central PMCID: PMCPMC6429627.

**3. The higher risk of clinical events within the first month in the high-intensity statin treated group points to the likely selection bias inherent in this sort of study design. Further supports the likelihood that cannot ascribe the clinical events observed to a treatment effect of the statin.**

**Answer :** As you pointed out, there was an inherent limitation of selection bias which was inevitable issue of observation study. As descripted in the manuscript, in acute phase of AMI, the ischemic complication (ie, cardiac death, MI or stent thrombosis etc…) frequently occurs in spite of optimal medical treatment including statin, because the status of patients suffering from AMI is still unstable and several factors may affect the early period of clinical outcomes. To minimize bias, we investigated monthly clinical events and performed the Cochran-Armitage trend test was used to determine difference in trends in incidence changes of event rates between acute and maintenance phase. As described in Appendix Figure 1, Cochran-Armitage trend testing showed that incidence rates were high in acute phase (<1 month) and decreased in maintenance phase (from 1 month to 12 months) regarding TVF, CV death and TV-MI. So, we decided to perform 1-month landmark analysis. We could demonstrate that the efficacy of high-intensity statin for secondary prevention in overall and younger patients with 1-month landmark analysis.

**Original text :** Line 213, results

We also presented the monthly incidence rates of clinical outcomes in overall and high- or low-to-moderate-intensity groups to identify a trend between acute and maintenance phase (Appendix Figure 1). As described in Appendix Figure 1, Cochran-Armitage trend testing revealed these trends to be statistically significant, with p value less than 0.001 in terms of CV death and TV-MI.

**Modified text :**

We also investigated the monthly incidence rates of clinical outcomes in overall population to identify a discriminating trends between acute and maintenance phase (Appendix Figure 1). As described in Appendix Figure 1, Cochran-Armitage trend testing revealed these trends to be statistically significant in terms of CV death and TV-MI (p<0.001), which showed incidence rates were high in acute phase (<1 month) and decreased in maintenance phase (from 1 month to 12 months).

**Modified text :** supplemental material

**Appendix Figure 1. Monthly incidence rates for clinical outcomes.**

† p value by Cochran-Armitage trend testing between acute phase (<1 month) and maintenance phase (from 1 month to 12 months)

*incidence rate of clinical outcome at 1 month

**mean value of incidence rate of clinical outcome from 1 month to 12 months

TVF, target vessel failure; CV, cardiovascular; TV-MI, target-vessel myocardial infarction; TLR, target lesion revascularization.

**Original text :** Line 325, discussion

1) This was a non-randomized, observational registry study with inherent methodological limitations; thus, its overall findings must be considered as hypothesis generating only.

**Modified text :**

1) This was a non-randomized, observational registry study with inherent methodological limitations; thus, its overall findings must be considered as hypothesis generating only. However, even though an inherent limitation, we could demonstrate that the efficacy of high-intensity statin for secondary prevention in younger patients with 1-month landmark analysis. This finding is well in line with the evidence provided by the current guidelines,[12, 13] in which means the clinical relavance of our result.

**4. We have seen from PROSPER, PALM and other studies that statins are generally quite well tolerated in older adults. While there are concerns about polypharmacy, frailty and drug interactions, the benefits likely outweigh the risks of high-intensity statin treatment in older adults with known ASCVD.**

**Answer :** Thanks for reviewer's comment. There have been several evidences of tolerance of statin in elderly patients. We presented the proportion of patients who continued the same intensity of statin in both younger and elderly patients. We added this in 'result’ and 'discussion’ session.

**Original text :** Line 261, results

**Modified text :**

Subgroup analysis for clinical impact of statin-intensity among patients continuing statin-intensity

In order to further support the results of our study, we performed subgroup analysis with patients who were continuing the same intensity of statin. Because adherence and tolerance are important clinical aspects of medical treatment that affects clinical outcomes,[17] we investigated the maintenance proportion of the same intensity of statin during 1 year after index AMI in total of 6,566 patients. In Figure 2, in patients who received high-intensity statin at discharge, the proportion of patients who maintained the same intensity of statin was significantly lower in elderly patients than in younger patients (55.0% vs. 66.3%, p=0.0009), which was not shown in low-to-moderate-intensity statin group (99.1% vs. 98.1%, p=0.06). In other words, de-escalation of statin-intensity was frequently occurred in elderly patients treated with high-intensity statin.

**Original text :** Line 271, discussion

2) in acute phase, there were higher risk of adverse clinical events in the high-intensity statin group

**Modified text :**

2) in patients who received high-intensity statin at discharge, the proportion of patients who maintained the same intensity of statin was significantly lower in elderly patients than that of younger patients;

**Reviewer #2: 1. The change of the amount of statin during follow-up due to side effects or lower the cholesterol level further is very common but not showed in this study. Cholesterol level at follow-up and use of rate of ezetimibe or PCSK9 did not show. Those variables can affect the clinical outcomes.**

**Answer :** I appreciated with reviewer’s valuable comment. With response to your comment, we investigated the change of statin intensity during 1 year after index AMI with a total of 6,566 patients. In patients who received high-intensity statin at discharge, the proportion of patients who maintained the same intensity of statin during 1 year was significantly lower in elderly patients than in younger patients (55.0% vs. 66.3%, p=0.0009), which was not shown in less-intensive statin group (99.1% vs. 98.1%, p=0.06) (please see below figure). In other words, de-escalation of statin-intensity was frequently observed in elderly patients treated with high-intensity statin.

In overall and younger patients, patients who maintained high-intensity statin showed significantly better clinical outcome in terms of TVF (overall: HR 0.64, 95% CI 0.46-0.9, p=0.01; younger patients: HR 0.63, 95% CI 0.44-0.9, p=0.012). However, in elderly patients, the efficacy for reducing adverse clinical outcomes did not differ between continuous use of high- and low-to-moderate-intensity statin (HR 0.7, 95% CI 0.28-1.77, p=0.46) (please see below table). These findings were consistent with the main result of our study. However, the incidence rate of TVF was numerically lower in high-intensity statin group compared to low-to-moderate-intensity statin group. To overcome the small number of event rates, we extended a study period to 24 months, which showed a consistent result (Appendix Table 5).

However, unfortunately, there were no available data regarding side effects of statin or prescription of ezetimibe, which was limitation of our study. While, PCSK9 inhibitor was not widely used due to insurance problem in the study period. We did not show cholesterol level at follow-up because of missing data. We added this in the ‘Limitation’ session. Thanks.

| **TVF** | **Event Rates (n/%*)** | | **Multivariate adjusted**† | |
| --- | --- | --- | --- | --- |
|  | **Less intensive** | **High-intensity** | **HR** (95% CI)** | **P** |
| **At 1-12 months** |  |  |  |  |
| **Overall** | 285 (5.9) | 39 (3.8) | 0.64 (0.46-0.9) | 0.01 |
| **< 75 years old** | 238 (6.0) | 34 (3.8) | 0.63 (0.44-0.9) | 0.012 |
| **≥ 75 years old** | 47 (5.2) | 5 (3.8) | 0.7 (0.28-1.77) | 0.46 |
| **At 1-24 months** |  |  |  |  |
| **Overall** | 478 (9.8) | 76 (7.4) | 0.75 (0.59-0.96) | 0.02 |
| **< 75 years old** | 384 (9.7) | 66 (7.4) | 0.76 (0.59-0.99) | 0.044 |
| **≥ 75 years old** | 94 (10.5) | 10 (7.5) | 0.71 (0.37-1.37) | 0.305 |

†adjusted by covariates including diabetes mellitus, chronic renal disease, peripheral artery disease, atrial fibrillation, left ventricle ejection fraction, left main disease, and total stent number.
*Event rates were derived from the Kaplan-Meier estimates. Hazard ratio is the risk of high-intensity statin for clinical outcomes compared with that of less intensive statin.
**P value by log-rank test was less than 0.05.
HR, hazard ratio; CI, confidence interval; TVF, target vessel failure.

**Original text :** Line 261, results

**Modified text :**

Subgroup analysis for clinical impact of statin-intensity among patients continuing statin-intensity

In order to further support the results of our study, we performed subgroup analysis with patients who was continuing the same intensity of statin. Because adherence and tolerance are important clinical aspects of medical treatment that affects clinical outcomes,[17] we investigated the maintenance proportion of the same intensity of statin during 1 year with a total of 6,566 patients. In Figure 2, in patients who received high-intensity statin at discharge, the proportion of patients who maintained the same intensity of statin was significantly lower in elderly patients than in younger patients (55.0% vs. 66.3%, p=0.0009), which was not shown in less-intensive statin group (99.1% vs. 98.1%, p=0.06). In other words, de-escalation of statin-intensity was frequently observed in elderly patients treated with high-intensity statin.

**Figure 2. Age differences on maintenance proportion of statin-intensity.**

**Appendix Table 5. Subgroup analysis of event rates and hazard ratios for target-vessel failure among patients with continuing statin-intensity at 1 year.**

| **TVF** | **Event Rates (n/%*)** | | **Multivariate adjusted**† | |
| --- | --- | --- | --- | --- |
|  | **Less intensive** | **High-intensity** | **HR** (95% CI)** | **P** |
| **At 1-12 months** |  |  |  |  |
| **Overall** | 285 (5.9) | 39 (3.8) | 0.64 (0.46-0.9) | 0.01 |
| **< 75 years old** | 238 (6.0) | 34 (3.8) | 0.63 (0.44-0.9) | 0.012 |
| **≥ 75 years old** | 47 (5.2) | 5 (3.8) | 0.7 (0.28-1.77) | 0.46 |
| **At 1-24 months** |  |  |  |  |
| **Overall** | 478 (9.8) | 76 (7.4) | 0.75 (0.59-0.96) | 0.02 |
| **< 75 years old** | 384 (9.7) | 66 (7.4) | 0.76 (0.59-0.99) | 0.044 |
| **≥ 75 years old** | 94 (10.5) | 10 (7.5) | 0.71 (0.37-1.37) | 0.305 |

†adjusted by covariates including diabetes mellitus, chronic renal disease, peripheral artery disease, atrial fibrillation, left ventricle ejection fraction, left main disease, and total stent number.
*Event rates were derived from the Kaplan-Meier estimates. Hazard ratio is the risk of high-intensity statin for clinical outcomes compared with that of less intensive statin.
**P value by log-rank test was less than 0.05.
HR, hazard ratio; CI, confidence interval; TVF, target vessel failure.

**Original text :** Line 331, discussion

4) To verify deterioration of endogenous cholesterol synthesis with increasing age, it is necessary to comfirm the markers of cholesterol metabolism such as lathosterol, campesterol and sitosterol and their ratios to cholesterol. However, this registry did not identify those kinds of markers.

**Modified text :**

4) No data was available regarding side effects of statin, prescription of ezetimibe. And PCSK9 inhibitor was not widely used in the study period due to governmental insurance coverage.

**2. In this study did not showed combined outcomes between 0 to 1-month and 1 to 12-month together in manuscript, which seems to be more important.**

**Answer :** We already provided the result of combined outcomes in Appendix Table 1, which clinical outcomes were not different according to statin-intensity in overall population. So, we additionally analyzed with subgroup who was continuing the same statin-intensity at 1 year, which showed favor result for high-intensity statin in overall and younger patients (overall: HR 0.63, 95% CI 0.45-0.87, p=0.005; younger patients: HR 0.63, 95% CI 0.44-0.89, p=0.009). In elderly patients, the incidence rate of TVF was numerically lower in high-intensity statin group compared to low-to-moderate intensity statin group (HR 0.6, 95% CI 0.24-1.51, p=0.28). After 1-month landmark analysis, it showed consistent result. We provided this result in the manuscript and supplement.

**Original text :** Line 262, results

**Modified text :**

In subgroup analysis, patients who maintained high-intensity statin showed significantly better clinical outcome in terms of TVF in overall and younger patients (overall: HR 0.64, 95% CI 0.46-0.9, p=0.01; younger patients: HR 0.63, 95% CI 0.44-0.9, p=0.012). However, in elderly patients, the efficacy for reducing adverse clinical outcomes did not differ between continuous use of high- and low-to-moderate-intensity statin (HR 0.7, 95% CI 0.28-1.77, p=0.46). These findings were consistent with the main result of our study. However, the incidence rate of TVF was numerically lower in high-intensity statin group compared to low-to-moderate-intensity statin group. To overcome the small number of event rates, we extended a study period to 24 months, which showed a consistent result (Appendix Table 5).

**Appendix Table 5. Subgroup analysis of event rates and hazard ratios for target-vessel failure among patients with continuing statin-intensity at 1 year.**

| **TVF** | **Event Rates (n/%*)** | | **Multivariate adjusted**† | |
| --- | --- | --- | --- | --- |
|  | **Less intensive** | **High-intensity** | **HR** (95% CI)** | **P** |
| **At 1-12 months** |  |  |  |  |
| **Overall** | 285 (5.9) | 39 (3.8) | 0.64 (0.46-0.9) | 0.01 |
| **< 75 years old** | 238 (6.0) | 34 (3.8) | 0.63 (0.44-0.9) | 0.012 |
| **≥ 75 years old** | 47 (5.2) | 5 (3.8) | 0.7 (0.28-1.77) | 0.46 |
| **At 1-24 months** |  |  |  |  |
| **Overall** | 478 (9.8) | 76 (7.4) | 0.75 (0.59-0.96) | 0.02 |
| **< 75 years old** | 384 (9.7) | 66 (7.4) | 0.76 (0.59-0.99) | 0.044 |
| **≥ 75 years old** | 94 (10.5) | 10 (7.5) | 0.71 (0.37-1.37) | 0.305 |

†adjusted by covariates including diabetes mellitus, chronic renal disease, peripheral artery disease, atrial fibrillation, left ventricle ejection fraction, left main disease, and total stent number.
*Event rates were derived from the Kaplan-Meier estimates. Hazard ratio is the risk of high-intensity statin for clinical outcomes compared with that of less intensive statin.
**P value by log-rank test was less than 0.05.
HR, hazard ratio; CI, confidence interval; TVF, target vessel failure.

**3. This study compared TVF in high dose statin group of >75 was higher than low to moderate dose, even statistically insignificant. Explanations are needed.**

**Answer :** Thank you for valuable comment. Owing to the relatively limited number, some result of this study may not be sufficiently powered to compare between statin-intensity groups in elderly patients. Especially in elderly patients, high-intensity statin showed numerically higher incidence rates of TVF without statistical significance in maintenance phase. To minimize the bias, we additionally performed subgroup analysis with patients who were continuing the same statin-intensity during 1 year. As already answered in the response to your comment #1, in elderly patients with continuing high-intensity statin, the incidence rate of TVF was numerically lower compared to less-intensive statin group, which was consistent through extended study period of 24 months. Finally, to respond your comment, we added a text the limitation session as below.

**Original text :** Line 330, discussion

**Modified text :**

3) Owing to the relatively limited number, some result of this study may not be sufficiently powered to compare between statin-intensity groups. Especially in elderly patients, high-intensity statin showed numerically higher incidence rates of TVF without statistical significance in maintenance phase. To minimize the bias, we additionally performed subgroup analysis with patients who was continuing the same statin-intensity during 1 year. In elderly patients with continuing high-intensity statin, the incidence rate of TVF was numerically lower compared to less-intensive statin group, which was consistent through extended study period of 24 months.

**4. The positive results of this study are marginal, which is hard to lead to the conclusion**.

**Answer :** With rigorous PS matching and subgroup analysis, the positive results were consistent, which showed better clinical outcomes of high-intensity statin in overall and younger patients. Especially, with subgroup analysis with continuing statin-intensity group, the statistical significance was strong enough (p=0.01 in overall and p=0.012 in younger patients). Thanks.

**Modified text :** descripted in No #2.

**5. In discussion, page 14 line 20, 1-month seems to be change into 1-year.**

**Answer :** We divided in acute phase and maintenance phase to estimate accurate efficacy of statin therapy with minimizing inherent selection bias. So, 1-month seem to be correct.
