# Supplementary material for: Clinical impact of statin intensity according to age in patients with acute myocardial infarction
Source: PLoS One. 2022 Jun 15;17(6):e0269301. doi: 10.1371/journal.pone.0269301 (PMC9200343; doi:10.1371/journal.pone.0269301)
Supplement: Attachment — Submitted filename: Response to Reviewers_R2.docx [file pone.0269301.s004.docx]

**Revision Response Letter**

Ref. No: PONE-D-21-14585R2

Title: Clinical Impact of Statin Intensity According to Age in Patients with Acute Myocardial Infarction.

Dear Dr. Emily Chenette

Academic Editor of PLOS ONE

We thank you, the reviewers and editorial staff for the constructive and valuable comments regarding our manuscript. We now provide a revised manuscript for consideration of publication in PLOS ONE. We have carefully considered each comment and attached a point-by-point-response to the reviewers’ comments. We hope that the paper will be of substantial interest to the readership of PLOS ONE. All authors concur with the submission and the material has not been published previously or is not under consideration for publication elsewhere.

On behalf of the authors and with best regards

**Mahn-Won Park, M.D., Ph.D., Associate Professor**

Department of Cardiology, Daejeon St. Mary’s Hospital, College of Medicine,

The Catholic University of Korea

64, Daeheung-ro, Jung-gu, Daejeon, 34943, Korea

Phone: +82-10-3800-2817, E-mail: pmw6193@catholic.ac.kr

We thank to the editor and reviewers for their time and input, and their precious comments.

**Journal Requirements:**

**1. Please review your reference list to ensure that it is complete and correct. If you have cited papers that have been retracted, please include the rationale for doing so in the manuscript text, or remove these references and replace them with relevant current references. Any changes to the reference list should be mentioned in the rebuttal letter that accompanies your revised manuscript. If you need to cite a retracted article, indicate the article’s retracted status in the References list and also include a citation and full reference for the retraction notice.**

**Answer :** Thank you for your encouraging comments and generous understanding of our study. We have tried our best to address the issues you pointed out. Thank you. We conducted an in-depth review of the references and strictly checked. Fortunately, there was no retracted article but, typo errors, which were all corrected.

**Modified text :**

**References**

9.	Oliveira JS, Pinheiro MB, Fairhall N, Walsh S, Chesterfield Franks T, Kwok W, et al. Evidence on Physical Activity and the Prevention of Frailty and Sarcopenia Among Older People: A Systematic Review to Inform the World Health Organization Physical Activity Guidelines. Journal of physical activity & health. 2020;17(12):1247-1258. Epub 2020/08/12. doi: 10.1123/jpah.2020-0323. PubMed PMID: 32781432.

20.	Nanna MG, Navar AM, Wang TY, Mi X, Virani SS, Louie MJ, et al. Statin Use and Adverse Effects Among Adults >75 Years of Age: Insights From the Patient and Provider Assessment of Lipid Management (PALM) Registry. Journal of the American Heart Association. 2018;7(10):e008546. Epub 2018/05/10. doi: 10.1161/jaha.118.008546. PubMed PMID: 29739801; PubMed Central PMCID: PMC6015311.

26.	Cholesterol Treatment Trialists' Collaboration. Efficacy and safety of statin therapy in older people: a meta-analysis of individual participant data from 28 randomised controlled trials. Lancet (London, England). 2019;393(10170):407-15. Epub 2019/02/05. doi: 10.1016/s0140-6736(18)31942-1. PubMed PMID: 30712900; PubMed Central PMCID: PMC6429627.

28.	Bach RG, Cannon CP, Giugliano RP, White JA, Lokhnygina Y, Bohula EA, et al. Effect of Simvastatin-Ezetimibe Compared With Simvastatin Monotherapy After Acute Coronary Syndrome Among Patients 75 Years or Older: A Secondary Analysis of a Randomized Clinical Trial. JAMA cardiology. 2019;4(9):846-54. Epub 2019/07/18. doi: 10.1001/jamacardio.2019.2306. PubMed PMID: 31314050; PubMed Central PMCID: PMC6647004.

**Additional Editor Comments (if provided):**

**The authors have addressed all comments and questions posed by the reviewers, however the manuscript requires a thorough revision for proper English diction and syntax. The authors should carefully review and edit their manuscript to make it clear and concise for the reader.**

**Answer :** We performed a thorough revision for proper English diction and syntax. To prove this, we submitted the ‘Editing Certificate’ of American Journal Experts. The modified parts of text are highlighted in blue so that you can recognize easily.

**Modified text :**

Line 64

Meanwhile, the efficacy to prevent adverse clinical events between high- and low-to-moderate-intensity statin was not statistically different in elderly patients. Further randomized study with large elderly population is warranted.

Line 119

We compared the efficacy on clinical outcomes according to statin-intensity in younger (<75 years old) and elderly (≥75 years old) patients, respectively.

Line 210

Adverse clinical outcomes were similar between two statin-intensity groups except for TLR,

Line 216

As described in Appendix Figure 1, Cochran-Armitage trend testing revealed these trends to be statistically significant in terms of CV death and TV-MI (p<0.001), which showed high incidence rates in acute phase (<1 month) and decreased trend in maintenance phase (from 1 month to 12 months).

Line 270

In high-intensity statin group, the proportion of patients continuing the same statin-intensity was significantly lower in elderly patients than that of younger patients (55.0% vs. 66.3%, p=0.0009), while this phenomenon was not observed in low-to-moderate-intensity statin group (99.1% vs. 98.1%, p=0.06) (Figure 2).

Line 294

2) in high-intensity statin group, the proportion of patients continuing the same statin-intensity was significantly lower in elderly patients than that of younger patients;

Line 332

IDEAL (Incremental DEcrease through Aggressive Lipid Lowering) Study randomly assigned 8,888 patients age ≤ 80 years with previous myocardial infarction to receive either intensive therapy with atorvastatin 80 mg/day or the standard statin therapy (simvastatin 20 to 40 mg/day) to compare the ability of these regimens to lower cardiovascular risk.

Line 342

Although some analyses reported an association between high-intensity statin use and a survival benefit in older adults,[25, 26] the study population was heterogenous while our study population consisted of only AMI patients. In one retrospective cohort analysis, even about 60% of CAD patients were included in the study population, there is no information of severity or revascularization of coronary lesions.[25] Besides, in the meta-analysis by Cholesterol Treatment Trialists’ Collaboration, 26 trials were based on analysis between statin therapy versus control, not comparison of statin-intensity.[26]

Line 368

However, even though there is an inherent limitation, we could demonstrate the efficacy of high-intensity statin for secondary prevention in younger patients. This finding is well in line with the evidence provided by the current guidelines.[12, 13]

Line 385

4) No data was available regarding side effects of statin and prescription rates of ezetimibe or PCSK9 inhibitor. Especially, PCSK9 inhibitor was not widely used in the study period due to governmental insurance coverage.

Line 396

Meanwhile, the efficacy to prevent adverse clinical events between high- and low-to-moderate-intensity statin was not statistically different in elderly patients. Further randomized study with large elderly population is warranted.
