# Supplementary material for: Clinical impact of statin intensity according to age in patients with acute myocardial infarction
Source: PLoS One. 2022 Jun 15;17(6):e0269301. doi: 10.1371/journal.pone.0269301 (PMC9200343; doi:10.1371/journal.pone.0269301)
Supplement: Attachment — Submitted filename: Response to Reviewers_R3.docx [file pone.0269301.s005.docx]

**Revision Response Letter**

Thank you for your encouraging comments and generous understanding of our study. We have tried our best to address the issues you pointed out. Thank you.

**REVIEWER #3 :**

**1. The authors review of the literature is that there is limited data to guide treatment decisions post-AMI in older adults, however this should be tempered and the rationale for this study should be clarified. The cited guidelines have the greatest ambiguity in relation to primary prevention, not statin use after AMI where time to benefit is short – on the order of days/months, not years as in primary prevention. The recommendation for lower-intensity statins in older adults is not strong and is based on only perceived risk of adverse effects (Mach EHJ 2020).**

Answer : We appreciate your valuable comment. Through the in-depth review of references, we modified original references into more suitable references. We also tried our best to temper and clarify the rationale of our study as follows;

**Original text : Background, Line 76**

Thus, current guidelines recommend that high-intensity statin should be considered based on lowering low-density lipoprotein cholesterol (LDL-C) for secondary prevention in patients with AMI.

Especially, because of globally increasing number of adults aged 75 years or older and inherent their high cardiovascular risk and frailty,

**Modified text :**

Thus, current guidelines recommend that high-intensity statin should be considered based on lowering low-density lipoprotein cholesterol (LDL-C) for secondary prevention in patients with atherosclerotic cardiovascular disease (ASCVD).

Because of globally increasing number of adults aged 75 years or older and inherent their high cardiovascular risk and frailty,

**Original text : Background, Line 99**

Therefore, to address this issue, we sought to compare the clinical efficacy of high-intensity versus low-to-moderate-intensity statin according to age (<75 or ≥75 years old) using large prospective AMI registry.

**Modified text :**

Therefore, we sought to (1) investigate the prescription rate of high-intensity statin and (2) compare the association between statin-intensity (high-intensity versus low-to-moderate-intensity statin) and clinical outcomes according to age (<75 or ≥75 years old) using large prospective AMI registry.

**Original text : References, Line 410**

1.	Ridker PM. The JUPITER trial: results, controversies, and implications for prevention. Circulation Cardiovascular quality and outcomes. 2009;2(3):279-85. Epub 2009/12/25. doi: 10.1161/circoutcomes.109.868299. PubMed PMID: 20031849.

**Modified text :**

1.	Collet JP, Thiele H, Barbato E, Barthélémy O, Bauersachs J, Bhatt DL, et al. 2020 ESC Guidelines for the management of acute coronary syndromes in patients presenting without persistent ST-segment elevation. European heart journal. 2021;42(14):1289-367. Epub 2020/08/30. doi: 10.1093/eurheartj/ehaa575. PubMed PMID: 32860058.

**Original text : References, Line 413**

2.	Baigent C, Blackwell L, Emberson J, Holland LE, Reith C, Bhala N, et al. Efficacy and safety of more intensive lowering of LDL cholesterol: a meta-analysis of data from 170,000 participants in 26 randomised trials. Lancet (London, England). 2010;376(9753):1670-81. Epub 2010/11/12. doi: 10.1016/s0140-6736(10)61350-5. PubMed PMID: 21067804; PubMed Central PMCID: PMC2988224.

3.	Fulcher J, O'Connell R, Voysey M, Emberson J, Blackwell L, Mihaylova B, et al. Efficacy and safety of LDL-lowering therapy among men and women: meta-analysis of individual data from 174,000 participants in 27 randomised trials. Lancet (London, England). 2015;385(9976):1397-405. Epub 2015/01/13. doi: 10.1016/s0140-6736(14)61368-4. PubMed PMID: 25579834.

4.	Mills EJ, Rachlis B, Wu P, Devereaux PJ, Arora P, Perri D. Primary prevention of cardiovascular mortality and events with statin treatments: a network meta-analysis involving more than 65,000 patients. Journal of the American College of Cardiology. 2008;52(22):1769-81. Epub 2008/11/22. doi: 10.1016/j.jacc.2008.08.039. PubMed PMID: 19022156.

**Modified text :**

2.	Amarenco P, Bogousslavsky J, Callahan A, 3rd, Goldstein LB, Hennerici M, Rudolph AE, et al. High-dose atorvastatin after stroke or transient ischemic attack. The New England journal of medicine. 2006;355(6):549-59. Epub 2006/08/11. doi: 10.1056/NEJMoa061894. PubMed PMID: 16899775.

3.	Baigent C, Blackwell L, Emberson J, Holland LE, Reith C, Bhala N, et al. Efficacy and safety of more intensive lowering of LDL cholesterol: a meta-analysis of data from 170,000 participants in 26 randomised trials. Lancet (London, England). 2010;376(9753):1670-81. Epub 2010/11/12. doi: 10.1016/s0140-6736(10)61350-5. PubMed PMID: 21067804; PubMed Central PMCID: PMCPMC2988224.

**2. More importantly, the choice of methods are unclear and make the interpretation of the findings challenging – what is the rationale for running crude models or simple multivariate models? It is well established in pharmacoepidemiology, specifically in statin research, that these methods are insufficient – those who are prescribed a statin are likely at higher risk of an event, exactly as is shown in this study. Confounding by indication must be accounted for and propensity score methods should be the primary analysis. See for example: Seeger JD, Kurth T, Walker AM. Use of propensity score technique to account for exposure-related covariates: an example and lesson. Med Care. 2007;45(10)(suppl 2):S143-S148. doi:10.1097/MLR.0b013e318074ce79**

**Additionally, the choice of using matching for the PS should be justified. This excludes all patients who could not be matched and may bias the results. See Elze MC, Gregson J, Baber U, et al. Comparison of propensity score methods and covariate adjustment: evaluation in 4 cardiovascular studies. J Am Coll Cardiol. 2017;69(3):345-357. doi:10.1016/j.jacc.2016.10.060 for alternatives and consider IPTW or Overlap Weighting methods that make use of the whole cohort.**

Answer : We appreciate your valuable comment. As you recommended, we performed IPTW analysis for the primary analysis in order to minimize the bias. And, we added the results in a main text and changed tables and figures as follows. Thanks again.

**Original text : Methods, Line 172**

For all crude, multivariable-adjusted analyses, treatment effects were evaluated in all the patients and in each group of younger and elderly patients.

**Modified text :**

Inverse probability of treatment weighting (IPTW) based on the propensity score (probability of receiving high-intensity statin) was used as the primary tool to adjust for differences in the baseline characteristics between the high-intensity and low-to-moderate intenstiy groups. Once each patient’s propensity score was estimated, weights were calculated using the method described in the previous literature.[16] We examined the similarity of the baseline characteristics between the treatment groups before and after IPTW.[17] After confirming the comparability of the two groups in the data with IPTW, we ran the Cox proportional hazard model and made statistical inference using robust standard errors (Huber sandwich estimator).[18]

For all crude, multivariable-adjusted, and IPTW analyses, treatment effects were evaluated in overall patients and in each group of younger and elderly patients.

**Original text : Results, Line 205**

Involvement of proximal left anterior descending artery was shown frequently in high-intensity statin group among younger patients (Table 1).

**Modified text :**

Involvement of proximal left anterior descending artery was shown frequently in high-intensity statin group among younger patients (Table 1). As shown in Table 2 and Appendix Table 2, majority of those differences of covariates were balanced after PS matching and IPTW.

**Original text : Tables**

**Modified text :**

**Table 2. Demographics after inverse probability weighting.**

|  | **<75 years old** | | | | **≥75 years old** | | | |
| --- | --- | --- | --- | --- | --- | --- | --- | --- |
| **Characteristics** | **Less intensive (n = 6133)** | **High-intensity (n = 5683)** | **P** | **SMD** | **Less-intensity (n = 1548)** | **High-intensity (n = 1435)** | **P** | **SMD** |
| **Baseline patients characteristics** | | | | | | | | |
| Age (years) | 58.5 ± 10.2 | 58.3 ± 9.8 | 0.64 | 0.015 | 80.1 ± 5.4 | 80.0 ± 5.1 | 0.76 | 0.019 |
| Male | 4878 (79.5) | 4592 (80.8) | 0.33 | 0.032 | 733 (47.4) | 691 (48.1) | 0.81 | 0.016 |
| Hypertension | 2896 (47.2) | 2689 (47.3) | 0.96 | 0.002 | 1012 (65.4) | 915 (63.8) | 0.61 | 0.034 |
| Diabetes mellitus | 1841 (30.0) | 1687 (29.7) | 0.82 | 0.007 | 505 (32.6) | 470 (32.8) | 0.97 | 0.003 |
| Hyperlipidemia | 1068 (17.4) | 1137 (20.0) | 0.039 | 0.066 | 192 (12.4) | 212 (14.8) | 0.32 | 0.068 |
| Current smoker | 2994 (48.8) | 2789 (49.1) | 0.88 | 0.005 | 235 (15.2) | 207 (14.4) | 0.74 | 0.021 |
| Clinical diagnosis |  |  | 0.55 | 0.019 |  |  | 0.99 | 0.002 |
| STEMI | 3417 (55.7) | 3111 (54.7) |  |  | 712 (46.0) | 659 (45.9) |  |  |
| NSTEMI | 2717 (44.3) | 2573 (45.3) |  |  | 836 (54.0) | 777 (54.1) |  |  |
| CKD | 99.3 (1.6) | 87.5 (1.5) | 0.84 | 0.006 | 19 (1.2) | 18 (1.3) | 0.96 | 0.003 |
| Prior MI | 156 (2.5) | 135 (2.4) | 0.77 | 0.011 | 72 (4.6) | 62 (4.4) | 0.84 | 0.014 |
| Prior PCI | 290 (4.7) | 266 (4.7) | 0.94 | 0.002 | 127 (8.2) | 116 (8.1) | 0.95 | 0.004 |
| Prior CABG | 19 (0.3) | 10 (0.2) | 0.58 | 0.027 | 11 (0.7) | 11 (0.8) | 0.90 | 0.009 |
| Prior CVA | 350 (5.7) | 323 (5.7) | 0.97 | 0.001 | 158 (10.2) | 142 (9.9) | 0.86 | 0.011 |
| PAD | 24 (0.4) | 24 (0.4) | 0.90 | 0.005 | 12 (0.8) | 10 (0.7) | 0.92 | 0.009 |
| AF | 121 (2.0) | 93 (1.6) | 0.53 | 0.025 | 70 (4.5) | 54 (3.8) | 0.65 | 0.039 |
| LV EF, % | 54.2 ± 10.5 | 54.2 ± 10.7 | 0.83 | 0.007 | 51.0 ± 11.9 | 50.7 ± 11.6 | 0.67 | 0.028 |
| **Lesion and Procedural characteristics** | | | | | | | | |
| Radial access | 1244 (20.3) | 1212 (21.3) | 0.40 | 0.026 | 343 (22.1) | 333 (23.2) | 0.71 | 0.027 |
| LM involved | 353 (5.8) | 336 (5.9) | 0.84 | 0.006 | 139 (9.0) | 118 (8.2) | 0.70 | 0.028 |
| pLAD involved | 2541 (41.4) | 2500 (44.0) | 0.11 | 0.052 | 707 (45.7) | 693 (48.3) | 0.44 | 0.051 |
| Disease extent |  |  | 0.44 | 0.052 |  |  | 0.35 | 0.12 |
| 1VD | 2939 (47.9) | 2715 (47.8) |  |  | 572 (37.0) | 607 (42.3) |  |  |
| 2VD | 1966 (32.1) | 1870 (32.9) |  |  | 539 (34.8) | 463 (32.2) |  |  |
| 3VD | 1228 (20.0) | 1099 (19.3) |  |  | 437 (28.2) | 366 (25.5) |  |  |
| Complex PCI | 2636 (43.0) | 2484 (43.7) | 0.65 | 0.015 | 705 (45.6) | 665 (46.3) | 0.82 | 0.015 |
| Total stent number | 1.62 ± 0.88 | 1.64 ± 0.91 | 0.47 | 0.025 | 1.67 ± 0.85 | 1.66 ± 0.90 | 0.87 | 0.012 |
| Mean stent diameter | 3.20 ± 0.42 | 3.20 ± 0.51 | 0.97 | 0.001 | 3.07 ± 0.35 | 3.07 ± 0.77 | 0.94 | 0.006 |
| Total stent length | 34.47 ± 20.97 | 35.20 ± 22.73 | 0.34 | 0.033 | 35.65 ± 20.66 | 34.97 ± 21.53 | 0.65 | 0.032 |
| IVUS use | 1382 (22.5) | 1279 (22.5) | 0.98 | 0.001 | 261 (16.8) | 234 (16.3) | 0.82 | 0.015 |

SMD, standardised mean difference; STEMI, ST-segment elevation myocardial infarction; NSTEMI, non ST-segment elevation myocardial infarction; CKD, chronic kidney disease; MI, myocardial infarction; PCI, percutaneous coronary intervention; CABG, coronary artery bypass grafting surgery; CVA, cerebrovascular attack; PAD, peripheral artery disease; AF, atrial fibrillation; LV EF, left ventricle ejection fraction; HDL, high-density lipoprotein; LDL, low-density lipoprotein; LM, left main; pLAD, proximal left anterior descending artery; VD, vessel disease; IVUS, intravascular ultrasound.

**Original text : Table 2, 3**

**Table 2.** Event rates and hazard ratios for clinical outcomes in acute phase (<1 month).

| **Outcomes** | **Event Rates at 1 Month (n/%*)** | | **Crude** | | **Multivariate Adjusted**† | |  |
| --- | --- | --- | --- | --- | --- | --- | --- |
|  | **Low-to-moderate-intensity** | **High-intensity** | **HR (95% CI)** | **P** | **HR (95% CI)** | **P** | **P_Interaction_** |
| **Overall** |  |  |  |  |  |  |  |
| TVF | 52 (0.8)** | 25 (1.3)** | 1.6 (1.0-2.58) | 0.052 | 1.72 (1.07-2.78) | 0.026 | 0.76 |
| All-cause death | 28 (0.5)** | 17 (0.9)** | 2.03 (1.11-3.7) | 0.022 | 2.21 (1.21-4.04) | 0.01 | 0.91 |
| CV death | 27 (0.4)** | 16 (0.9)** | 1.98 (1.07-3.67) | 0.031 | 2.16 (1.16-4.02) | 0.015 | 0.65 |
| TV-MI | 10 (0.2) | 6 (0.3) | 2.0 (0.73-5.49) | 0.18 | 2.07 (0.75-5.71) | 0.16 | 0.52 |
| TLR | 23 (0.4) | 5 (0.3) | 0.72 (0.27-1.9) | 0.51 | 0.76 (0.29-2.0) | 0.58 | 0.46 |
| **< 75 years old** |  |  |  |  |  |  |  |
| TVF | 31 (0.6) | 16 (1.1) | 1.68 (0.92-3.07) | 0.09 | 1.84 (1.00-3.37) | 0.048 |  |
| All-cause death | 16 (0.3) | 10 (0.7) | 2.04 (0.92-4.49) | 0.08 | 2.34 (1.06-5.17) | 0.036 |  |
| CV death | 15 (0.3) | 10 (0.7) | 2.17 (0.98-4.84) | 0.057 | 2.49 (1.12-5.56) | 0.026 |  |
| TV-MI | 8 (0.2) | 4 (0.3) | 1.63 (0.49-5.4) | 0.43 | 1.72 (0.52-5.37) | 0.38 |  |
| TLR | 14 (0.3) | 4 (0.3) | 0.93 (0.31-2.82) | 0.90 | 0.99 (0.32-3.01) | 0.98 |  |
| **≥ 75 years old** |  |  |  |  |  |  |  |
| TVF | 21 (1.7) | 9 (2.6) | 1.57 (0.72-3.44) | 0.26 | 1.57 (0.72-3.42) | 0.26 |  |
| All-cause death | 12 (0.9) | 7 (2.0) | 2.14 (0.84-5.44) | 0.11 | 2.13 (0.84-5.4) | 0.11 |  |
| CV death | 12 (0.9) | 6 (1.7) | 1.84 (0.69-4.89) | 0.22 | 1.82 (0.68-4.86) | 0.23 |  |
| TV-MI | 2 (0.2) | 2 (0.6) | 3.65 (0.51-25.9) | 0.20 | 3.63 (0.51-25.8) | 0.20 |  |
| TLR | 9 (0.7) | 1 (0.3) | 0.41 (0.05-3.2) | 0.39 | 0.41 (0.05-3.25) | 0.40 |  |

†adjusted by covariates including age, diabetes mellitus.
*Event rates were derived from the Kaplan-Meier estimates. Hazard ratio is the risk of high-intensity statin for clinical outcomes compared with that of less intensive statin.
**P value by log-rank test was less than 0.05.
HR, hazard ratio; TVF, target vessel failure; CV, cardiovascular; TV-MI, target-vessel myocardial infarction; TLR, target lesion revascularization; ST, stent thrombosis.

**Table 3.** Event rates and hazard ratios for clinical outcomes in maintenance phase (from 1 month to 12 months).

| **Outcomes** | **Event Rates at 1-12 Month (n/%*)** | | **Crude** | | **Multivariate Adjusted**† | |  |
| --- | --- | --- | --- | --- | --- | --- | --- |
|  | **Low-to-moderate-intensity** | **High-intensity** | **HR (95% CI)** | **P** | **HR (95% CI)** | **P** | **P_interaction_** |
| **Overall** |  |  |  |  |  |  |  |
| TVF | 460 (7.7) | 117 (6.6) | 0.86 (0.7-1.05) | 0.14 | 0.85 (0.69-1.05) | 0.12 | 0.10 |
| All-cause death | 220 (3.7) | 71 (4.0) | 1.1 (0.84-1.43) | 0.50 | 1.15 (0.87-1.52) | 0.33 | 0.20 |
| CV death | 164 (2.7) | 53 (3.0) | 1.1 (0.8-1.49) | 0.56 | 1.1 (0.79-1.53) | 0.58 | 0.15 |
| TV-MI | 38 (0.6) | 10 (0.6) | 0.89 (0.45-1.79) | 0.75 | 0.83 (0.4-1.73) | 0.62 | >0.99 |
| TLR | 295 (5.0)** | 64 (3.7)** | 0.73 (0.56-0.96) | 0.024 | 0.73 (0.55-0.96) | 0.022 | 0.78 |
| **< 75 years old** |  |  |  |  |  |  |  |
| TVF | 329 (6.9) | 79 (5.5) | 0.79 (0.62-1.01) | 0.059 | 0.76 (0.59-0.99) | 0.038 |  |
| All-cause death | 107 (2.2) | 32 (2.2) | 0.99 (0.67-1.47) | 0.95 | 0.97 (0.63-1.49) | 0.89 |  |
| CV death | 76 (1.6) | 22 (1.5) | 0.96 (0.59-1.54) | 0.85 | 0.85 (0.5-1.46) | 0.56 |  |
| TV-MI | 27 (0.6) | 10 (0.7) | 1.22 (0.59-2.53) | 0.59 | 1.11 (0.52-2.39) | 0.79 |  |
| TLR | 250 (5.3)** | 55 (3.9)** | 0.72 (0.54-0.97) | 0.029 | 0.71 (0.53-0.96) | 0.025 |  |
| **≥ 75 years old** |  |  |  |  |  |  |  |
| TVF | 131 (10.8) | 38 (11.7) | 1.1 (0.76-1.58) | 0.61 | 1.1 (0.76-1.59) | 0.63 |  |
| All-cause death | 113 (9.2) | 39 (11.8) | 1.32 (0.91-1.89) | 0.14 | 1.35 (0.92-1.97) | 0.12 |  |
| CV death | 88 (7.2) | 31 (9.5) | 1.34 (0.89-2.02) | 0.16 | 1.34 (0.88-2.05) | 0.18 |  |
| TV-MI | 11 (0.9) | 0 | NA | >0.99 | NA | >0.99 |  |
| TLR | 45 (3.9) | 9 (2.9) | 0.76 (0.37-1.55) | 0.45 | 0.76 (0.37-1.57) | 0.46 |  |

†adjusted by covariates including diabetes mellitus, chronic renal disease, peripheral artery disease, atrial fibrillation, left ventricle ejection fraction, left main disease, and total stent number.
*Event rates were derived from the Kaplan-Meier estimates. Hazard ratio is the risk of high-intensity statin for clinical outcomes compared with that of less intensive statin.
**P value by log-rank test was less than 0.05.
HR, hazard ratio; TVF, target vessel failure; CV, cardiovascular; TV-MI, target-vessel myocardial infarction; TLR, target lesion revascularization.

**Modified text :**

**Table 3.** Event rates and hazard ratios for clinical outcomes in acute phase (<1 month).

| **Outcomes** | **Event Rates at 1 Month (n/%*)** | | **Crude** | | **Multivariate Adjusted**† | | **IPTW Adjusted** | | |  |
| --- | --- | --- | --- | --- | --- | --- | --- | --- | --- | --- |
|  | **Low-to-moderate-intensity** | **High-intensity** | **HR (95% CI)** | **P** | **HR (95% CI)** | **P** | **HR (95% CI)** | **P** | **P_Interaction_** |  |
| **Overall** |  |  |  |  |  |  |  |  |  |  |
| TVF | 52 (0.8)** | 25 (1.3)** | 1.6 (1.0-2.58) | 0.052 | 1.72 (1.07-2.78) | 0.026 | 1.24 (0.73-2.10) | 0.43 | 0.97 |  |
| All-cause death | 28 (0.5)** | 17 (0.9)** | 2.03 (1.11-3.7) | 0.022 | 2.21 (1.21-4.04) | 0.01 | 1.37 (0.70-2.70) | 0.36 | 0.96 |  |
| CV death | 27 (0.4)** | 16 (0.9)** | 1.98 (1.07-3.67) | 0.031 | 2.16 (1.16-4.02) | 0.015 | 1.32 (0.65-2.66) | 0.44 | 0.68 |  |
| TV-MI | 10 (0.2) | 6 (0.3) | 2.0 (0.73-5.49) | 0.18 | 2.07 (0.75-5.71) | 0.16 | 1.72 (0.61-4.86) | 0.31 | 0.31 |  |
| TLR | 23 (0.4) | 5 (0.3) | 0.72 (0.27-1.9) | 0.51 | 0.76 (0.29-2.0) | 0.58 | 0.65 (0.24-1.78) | 0.40 | 0.73 |  |
| **< 75 years old** |  |  |  |  |  |  |  |  |  | |
| TVF | 31 (0.6) | 16 (1.1) | 1.68 (0.92-3.07) | 0.09 | 1.84 (1.00-3.37) | 0.048 | 1.23 (0.63-2.38) | 0.54 |  | |
| All-cause death | 16 (0.3) | 10 (0.7) | 2.04 (0.92-4.49) | 0.08 | 2.34 (1.06-5.17) | 0.036 | 1.39 (0.57-3.38) | 0.47 |  | |
| CV death | 15 (0.3) | 10 (0.7) | 2.17 (0.98-4.84) | 0.057 | 2.49 (1.12-5.56) | 0.026 | 1.48 (0.60-3.64) | 0.39 |  | |
| TV-MI | 8 (0.2) | 4 (0.3) | 1.63 (0.49-5.4) | 0.43 | 1.72 (0.52-5.37) | 0.38 | 1.16 (0.35-3.90) | 0.81 |  | |
| TLR | 14 (0.3) | 4 (0.3) | 0.93 (0.31-2.82) | 0.90 | 0.99 (0.32-3.01) | 0.98 | 0.73 (0.23-2.29) | 0.59 |  | |
| **≥ 75 years old** |  |  |  |  |  |  |  |  |  | |
| TVF | 21 (1.7) | 9 (2.6) | 1.57 (0.72-3.44) | 0.26 | 1.57 (0.72-3.42) | 0.26 | 1.21 (0.51-2.87) | 0.66 |  | |
| All-cause death | 12 (0.9) | 7 (2.0) | 2.14 (0.84-5.44) | 0.11 | 2.13 (0.84-5.4) | 0.11 | 1.37 (0.46-4.04) | 0.57 |  | |
| CV death | 12 (0.9) | 6 (1.7) | 1.84 (0.69-4.89) | 0.22 | 1.82 (0.68-4.86) | 0.23 | 1.08 (0.34-3.37) | 0.90 |  | |
| TV-MI | 2 (0.2) | 2 (0.6) | 3.65 (0.51-25.9) | 0.20 | 3.63 (0.51-25.8) | 0.20 | 3.92 (0.5-30.53) | 0.19 |  | |
| TLR | 9 (0.7) | 1 (0.3) | 0.41 (0.05-3.2) | 0.39 | 0.41 (0.05-3.25) | 0.40 | 0.48 (0.07-3.61) | 0.48 |  | |

†adjusted by covariates including age, diabetes mellitus.
*Event rates were derived from the Kaplan-Meier estimates. Hazard ratio is the risk of high-intensity statin for clinical outcomes compared with that of less intensive statin.
**P value by log-rank test was less than 0.05.
HR, hazard ratio; TVF, target vessel failure; CV, cardiovascular; TV-MI, target-vessel myocardial infarction; TLR, target lesion revascularization.

**Table 4.** Event rates and hazard ratios for clinical outcomes in maintenance phase (from 1 month to 12 months).

| **Outcomes** | **Event Rates at 1-12 Month (n/%*)** | | **Crude** | | **Multivariate Adjusted**† | | **IPTW Adjusted** | | |  |
| --- | --- | --- | --- | --- | --- | --- | --- | --- | --- | --- |
|  | **Low-to-moderate-intensity** | **High-intensity** | **HR (95% CI)** | **P** | **HR (95% CI)** | **P** | **HR (95% CI)** | **P** | **P_Interaction_** |  |
| **Overall** |  |  |  |  |  |  |  |  |  |  |
| TVF | 460 (7.7) | 117 (6.6) | 0.86 (0.7-1.05) | 0.14 | 0.85 (0.69-1.05) | 0.12 | 0.85 (0.68-1.06) | 0.15 | 0.17 |  |
| All-cause death | 220 (3.7) | 71 (4.0) | 1.1 (0.84-1.43) | 0.50 | 1.15 (0.87-1.52) | 0.33 | 1.17 (0.86-1.57) | 0.32 | 0.30 |  |
| CV death | 164 (2.7) | 53 (3.0) | 1.1 (0.8-1.49) | 0.56 | 1.1 (0.79-1.53) | 0.58 | 1.06 (0.75-1.49) | 0.76 | 0.28 |  |
| TV-MI | 38 (0.6) | 10 (0.6) | 0.89 (0.45-1.79) | 0.75 | 0.83 (0.4-1.73) | 0.62 | 0.84 (0.37-1.92) | 0.69 | NA |  |
| TLR | 295 (5.0)** | 64 (3.7)** | 0.73 (0.56-0.96) | 0.024 | 0.73 (0.55-0.96) | 0.022 | 0.72 (0.54-0.97) | 0.033 | 0.76 |  |
| **< 75 years old** |  |  |  |  |  |  |  |  |  | |
| TVF | 329 (6.9) | 79 (5.5) | 0.79 (0.62-1.01) | 0.059 | 0.76 (0.59-0.99) | 0.038 | 0.75 (0.57-0.99) | 0.044 |  | |
| All-cause death | 107 (2.2) | 32 (2.2) | 0.99 (0.67-1.47) | 0.95 | 0.97 (0.63-1.49) | 0.89 | 0.96 (0.61-1.52) | 0.86 |  | |
| CV death | 76 (1.6) | 22 (1.5) | 0.96 (0.59-1.54) | 0.85 | 0.85 (0.5-1.46) | 0.56 | 0.82 (0.47-1.43) | 0.48 |  | |
| TV-MI | 27 (0.6) | 10 (0.7) | 1.22 (0.59-2.53) | 0.59 | 1.11 (0.52-2.39) | 0.79 | 1.19 (0.51-2.76) | 0.69 |  | |
| TLR | 250 (5.3)** | 55 (3.9)** | 0.72 (0.54-0.97) | 0.029 | 0.71 (0.53-0.96) | 0.025 | 0.70 (0.51-0.97) | 0.033 |  | |
| **≥ 75 years old** |  |  |  |  |  |  |  |  |  | |
| TVF | 131 (10.8) | 38 (11.7) | 1.1 (0.76-1.58) | 0.61 | 1.1 (0.76-1.59) | 0.63 | 1.06 (0.72-1.57) | 0.76 |  | |
| All-cause death | 113 (9.2) | 39 (11.8) | 1.32 (0.91-1.89) | 0.14 | 1.35 (0.92-1.97) | 0.12 | 1.33 (0.90-1.97) | 0.15 |  | |
| CV death | 88 (7.2) | 31 (9.5) | 1.34 (0.89-2.02) | 0.16 | 1.34 (0.88-2.05) | 0.18 | 1.24 (0.80-1.92) | 0.35 |  | |
| TV-MI | 11 (0.9) | 0 | NA |  | NA |  | NA |  |  | |
| TLR | 45 (3.9) | 9 (2.9) | 0.76 (0.37-1.55) | 0.45 | 0.76 (0.37-1.57) | 0.46 | 0.81 (0.37-1.75) | 0.59 |  | |

†adjusted by covariates including age, diabetes mellitus.
*Event rates were derived from the Kaplan-Meier estimates. Hazard ratio is the risk of high-intensity statin for clinical outcomes compared with that of less intensive statin.
**P value by log-rank test was less than 0.05.
HR, hazard ratio; TVF, target vessel failure; CV, cardiovascular; TV-MI, target-vessel myocardial infarction; TLR, target lesion revascularization.

**Original text : Results, Line 222**

The incidence rates of clinical outcomes between two statin-intensity groups in the overall population, younger and elderly patients within 1 month after index PCI are shown in Table 2. Kaplan-Meier curves for TVF and TLR at 1 month were shown in Figure 3. In overall patients, early clinical outcomes regarding TVF, all-cause death and CV death were worse in the high-intensity statin group (TVF : aHR, 1.72; 95% CI, 1.07-2.78; p=0.026; all-cause death : aHR, 2.21; 95% CI, 1.21-4.04; p=0.01; CV death : aHR, 2.16; 95% CI, 1.16-4.02; p=0.015). After subgroup analysis by aged of 75, these findings were consistent in younger patients. However, in elderly patients, although there were numerically higher event rates in the high-intensity statin group without statistical significance. No significant interactions were found between aged group and statin intensity in any of the adjusted 1-month risks of study outcomes (P_interaction_ = 0.76 for TVF, P_interaction_ = 0.65 for CV death, and P_interaction_ = 0.46 for TLR).

**Modified text :**

The incidence rates of clinical outcomes between two statin-intensity groups in the overall population, younger and elderly patients within 1 month after index PCI are shown in Table 3. Kaplan-Meier curves for TVF at 1 month were shown in Figure 3, adjusted by IPTW analysis. In overall patients, the high-intensity statin group showed poorer 1-month clinical outcomes regarding TVF, all-cause death and CV death (TVF : aHR, 1.72; 95% CI, 1.07-2.78; p=0.026; all-cause death : aHR, 2.21; 95% CI, 1.21-4.04; p=0.01; CV death : aHR, 2.16; 95% CI, 1.16-4.02; p=0.015). However, after IPTW analysis, no differences were observed with statin-intensity in both younger and elderly patients (Table 3).

After subgroup analysis by aged of 75, these findings were consistent in younger patients. Meanwhile, in elderly patients, despite numerically higher event rates in the high-intensity statin group, there were no statistical significance (Table 3). No significant interactions were found between aged group and statin intensity in any of the adjusted 1-month risks of study outcomes (P_interaction_ = 0.97 for TVF, P_interaction_ = 0.96 for all-cause death, P_interaction_ = 0.68 for CV death, P_interaction_ = 0.31 for TV-MI and P_interaction_ = 0.73 for TLR).

**Original text : Results, Line 236**

In overall population, the risk of adverse clinical outcomes according to statin-intensity was not different except for TLR (TVF : aHR, 0.85; 95% CI, 0.69-1.05; p=0.12; all-cause death : aHR, 1.15; 95% CI, 0.87-1.52; p=0.33; TLR : aHR, 0.73; 95% CI, 0.55-0.96; p=0.022). In younger patients, the high-intensity statin group showed significantly better clinical outcomes in terms of TVF (aHR, 0.76; 95% CI, 0.59-0.99; p=0.038) and TLR (aHR, 0.72; 95% CI, 0.54-0.97; p=0.032) than the low-to-moderate-intensity statin group. However, intriguingly, in elderly patients, the efficacy for reducing adverse clinical outcomes between two statin-intensity groups did not differ (TVF : aHR, 1.1; 95% CI, 0.76-1.59; p=0.63; TLR : aHR, 0.76; 95% CI, 0.37-1.57; p=0.46) (Table 3).

The forest plot of hazard ratio for adverse clinical outcomes from 1 month to 12 months was shown in Figure 4. No significant treatment interactions were detected in subgroups defined by aged of 75 (P_interaction_ = 0.10 for TVF, P_interaction_ = 0.15 for CV death, and P_interaction_ = 0.78 for TLR). Kaplan-Meier curves for the secondary end points were also shown in Appendix Figure 2.

**Modified text :**

The incidence rates of clinical outcomes between two statin-intensity groups in the overall population, younger and elderly patients in maintenance phase are shown in Table 4. Kaplan-Meier curves for TVF and secondary end points in maintenance phase were shown in Figure 3 and Appendix Figure 2, which were adjusted by IPTW analysis. In overall population, the risk of adverse clinical outcomes according to statin-intensity was not different except for TLR (TVF : aHR, 0.85; 95% CI, 0.69-1.05; p=0.12; TLR : aHR, 0.73; 95% CI, 0.55-0.96; p=0.022; Table 4). In younger patients, the high-intensity statin group showed significantly better clinical outcomes in terms of TVF (aHR, 0.76; 95% CI, 0.59-0.99; p=0.038) and TLR (aHR, 0.72; 95% CI, 0.54-0.97; p=0.032) than the low-to-moderate-intensity statin group. However, intriguingly, in elderly patients, the efficacy for reducing adverse clinical outcomes between two statin-intensity groups did not differ (TVF : aHR, 1.1; 95% CI, 0.76-1.59; p=0.63; TLR : aHR, 0.76; 95% CI, 0.37-1.57; p=0.46). These findings were unchanged after IPTW adjustment for differences in baseline covariates.

The forest plot of hazard ratio after IPTW analysis for adverse clinical outcomes from 1 month to 12 months was shown in Figure 4. No significant treatment interactions were detected in subgroups defined by aged of 75 (P_interaction_ = 0.18 for TVF, P_interaction_ = 0.35 for all-cause death, P_interaction_ = 0.15 for CV death, and P_interaction_ = 0.76 for TLR).

**Original text : Figure 3**

**Modified text :**

**Figure 3.** Adjusted Kaplan-Meier curves for the primary end point according to statin-intensity in younger and elderly patients using inverse probability weighting.

**Original text : Results, Line 245.**

The forest plot of hazard ratio for adverse clinical outcomes from 1 month to 12 months was shown in Figure 4. No significant treatment interactions were detected in subgroups defined by aged of 75 (P_interaction_ = 0.10 for TVF, P_interaction_ = 0.15 for CV death, and P_interaction_ = 0.78 for TLR). Kaplan-Meier curves for the secondary end points were also shown in Appendix Figure 2.

**Modified text :**

The forest plot of hazard ratio after IPTW analysis for adverse clinical outcomes from 1 month to 12 months was shown in Figure 4. No significant treatment interactions were detected in subgroups defined by aged of 75 (P_interaction_ = 0.18 for TVF, P_interaction_ = 0.35 for all-cause death, P_interaction_ = 0.15 for CV death, and P_interaction_ = 0.76 for TLR).

**Original text : Figure legends, Line 550.**

**Modified text :**

**Figure 4.**

Effects of statin intensity on clinical outcomes in maintenance phase after IPTW analysis, subdivided by aged of 75.
*derived from unmatched population
**aHR, adjusted hazard ratio for high-intensity statin treatment compared with less intensive strategy after IPTW analysis.
TVF, target-vessel failure; TLR, target lesion revascularization; HR, hazard ratio; NA, not applicable.

**Figure 4.**

High-intensity Better

Low-to-moderate-intensity Better

**3. The primary outcome needs to be clarified – is it a composite of TVF, CV death, TV-MI, TLR, or are these co-primary? If they are co-primary, there needs to be correction for multiple testing. If it is a composite, the main results should be interpreted as the composite and only secondarily should individual findings be highlighted. Similarly, findings should be tempered accordingly.**

Answer : Thank you for your comment. In methods session, we clarified the primary outcome as a composite. As you recommended, we described primary and secondary outcomes separately in results session. To convey a clearer message, unnecessary secondary outcomes were omitted. Also, we changed the previous figure for K-M curves to make it easier for readers to understand.

**Original text : Methods, Line 133**

The primary end point of the study was target-vessel failure (TVF), defined as a composite of cardiovascular (CV) death, target-vessel MI (TV-MI), or target lesion revascularization (TLR). The secondary end points were included the individual components of primary end point, any death, any MI, any repeat revascularization (RR), patient-oriented clinical event (POCE), defined as a composite of any death, any MI, or any RR.

**Modified text :**

The primary end point of the study was target-vessel failure (TVF), defined as a composite of cardiovascular (CV) death, target-vessel MI (TV-MI), or target lesion revascularization (TLR). The secondary end points included the individual components of primary end point, and any death.

**Original text : Results, Line 224**

Kaplan-Meier curves for TVF and TLR at 1 month were shown in Figure 3.

**Modified text :**

Kaplan-Meier curves for TVF at 1 month were shown in Figure 3, adjusted by IPTW analysis.

**Original text : Appendix Figure 2.**

**Modified text :**

**Appendix Figure 2. Adjusted** **Kaplan-Meier curves for the secondary end points from 1 month to 12 months using inverse probability weighting.**

A-B) Cardiovascular death; C-D) Target vessel myocardial infarction; E-F) Target lesion revascularization.

Left panels (A,C,E) represented clinical outcomes in younger patients (<75 years old) and right panels (B,D,F) in elderly patients (≥75 years old).

CV, cardiovascular; TV-MI, target vessel myocardial infarction; TLR, target lesion revascularization.

**Minor: Avoid the use of trial language – this is an observational study and associations are being studied, not effects or efficacy.**

Answer : Thank you for your advice. We corrected the expression ‘effects or efficacy’ throughout the manuscript.

**Original text : Abstract, Line 51 and 63.**

**Modified text :**

**Methods** Using the COREA-AMI registry, we sought to compare the clinical impact of high- versus low-to-moderate-intensity statin in younger (<75 years old) and elderly (≥75 years old) patients. Of 10,719 patients, we included 8,096 patients treated with drug-eluting stents. All patients were classified into high-intensity versus low-to-moderate-intensity statin group according to statin type and dose at discharge. The primary end point was target-vessel failure (TVF), a composite of cardiovascular death, target-vessel MI, or target-lesion revascularization (TLR) from 1 month to 12 months after index PCI.

**Conclusions** In this AMI cohort underwent PCI, high-intensity statin showed the better 1-year clinical outcomes than low-to-moderate-intensity statin in younger patients. Meanwhile, the incidence rates of adverse clinical events between high- and low-to-moderate-intensity statin were not statistically different in elderly patients. Further randomized study with large elderly population is warranted.

**Original text : Results, Line 241.**

**Modified text :**

However, intriguingly, in elderly patients, the incidence rates of adverse clinical outcomes between two statin-intensity groups did not differ (TVF : aHR, 1.1; 95% CI, 0.76-1.59; p=0.63; TLR : aHR, 0.76; 95% CI, 0.37-1.57; p=0.46).

**Original text : Results, Line 277.**

**Modifed text :**

However, in elderly patients, the incidence rates of adverse clinical outcomes did not differ between continuous use of high- and low-to-moderate-intensity statin (aHR 0.7, 95% CI 0.28-1.77, p=0.46).

**Original text : Discussion, Line 298.**

**Modified text :**

4) in contrast, the incidence rates of adverse clinical events between high- and low-to-moderate-intensity statin were not statistically different in elderly patients.

**Original text : Conclusion, Line 396**

**Modified text :**

In this AMI cohort underwent PCI, high-intensity statin showed better clinical outcomes at 1-year than low-to-moderate intensity statin in younger patients. Meanwhile, in elderly patients, Meanwhile, the incidence rates of adverse clinical events were not statistically different between high- and low-to-moderate-intensity statin in elderly patients. Further randomized study with large elderly population is warranted.
