# Supplementary material for: Clinical impact of statin intensity according to age in patients with acute myocardial infarction
Source: PLoS One. 2022 Jun 15;17(6):e0269301. doi: 10.1371/journal.pone.0269301 (PMC9200343; doi:10.1371/journal.pone.0269301)
Supplement: Attachment — Submitted filename: RESPONSE_R4.docx [file pone.0269301.s006.docx]

**Reviewer #3: All my concerns have been adequately addressed.**

**This is a nice contribution to the literature.**

**ANSWER** : Thank you for your generous understanding. Thanks.

**Reviewer #4: The author analyzed clinical impact of statin intensity as secondary prevention in patients with acute myocardial infarction (AMI) using large, prospective, multicenter registry data. Patients were divided who treated percutaneous coronary intervention (PCI) due to AMI into taking high-intensity statin or low to moderate-intensity statin based on the age at which the 75-years old. The propensity score matching and inverse probability of treatment weighting methods were applied to properly compare the two groups, and as a consequence, the two groups could be compared relatively evenly. 1-year clinical outcomes showed that groups under 75 years old who took a high-intensity statin had better result than low-to-moderate-intensity statin group. However, in the groups which over 75-years old and overall groups show no significant difference according to intensity of statin doses. Although the characteristics of the patients subject to this study were heterogenous, comparison were made possible through appropriate statistical methods. The conclusion and discussion of this manuscript can help clinical direction in statin use as secondary preventions. However, there are several issues that should be adequately revised.

1. If patients have an LDL value during a 1-year follow-up periods, it will be feasible to determine how much the LDL level fluctuated for each statin group and explain the association by analyzing for a lowering trend in multiple event rates and LDLs.
 ANSWER** : Thank you for your valuable comment. We reviewed the raw data for LDL value during follow-up period. However, we found that 3,585 (44%) of follow-up LDL value were existed and the rest were missing value. Since most of follow-up LDL value were measured only once, we could not assess an LDL level fluctuation but could check a trend. In younger patients, high-intensity statin group showed more proportion of LDL level less than 70 mg/dL. In contrast, in elderly patients, there was no statistical difference according to statin-intensity. We could show that in the Table below. If the reviewer wants, it will be reflected in the manuscript.

|  | <75 years old | | | ≥75 years old | | |
| --- | --- | --- | --- | --- | --- | --- |
| Follow-up LDL-Cholesterol level (mg/dL) | Low to moderate intensity | High- intensity | p | Low to moderate intensity | High- intensity | p |
|  |  |  | <0.001 |  |  | 0.53 |
| ≥70 | 1418 (63.4) | 414 (48.6) |  | 218 (55.8) | 56 (52.3) |  |
| <70 | 818 (36.6) | 437 (51.4) |  | 173 (44.2) | 51 (47.7) |  |

**2. It would be more helpful to understand the background of the hypothesis to have an explanation or evidence for the criteria for dividing the age group by 75-years old.
 ANSWER** : I appreciate your comment. Statin therapy has been shown to reduce major vascular events and vascular mortality in patients with established ASCVD. However, rates of use of statin have been shown to decline with increasing age, and are substantially lower in elderly patients (> 75 years old), reflecting differences in both prescribing and compliance. One explanation might be that, there is a lack of data regarding the efficacy and safety of high-intensity statin in the elderly patients (> 75 years old), mainly because this patient group is often underrepresented in RCT comparing statin intensity. Also, current guidelines divided the age group by 75-years old. Therefore, we followed the definition of elderly patients as current guidelines did.

**Original text : Background, Line 92**

However, there is a lack of data regarding the efficacy and safety of high-intensity statin in the elderly patients (≥75 years old), mainly because this patient group is often underrepresented in randomised controlled trials comparing statin intensity.

**Modified text :**

However, prescription rates of statin have been shown to be declined with increasing age, and are lower, especially in elderly patients (>75 years old).[14] One explanation might be that, there has been a lack of data regarding the efficacy and safety of high-intensity statin in the elderly patients (≥75 years old), mainly because this patient group is often underrepresented in randomised controlled trials comparing statin intensity. For this reason, the level of evidence of using a high-intensity statin for elderly patients is lower compared to that for younger patients in current guidelines,[11, 12] of which elderly patients were divided the age goup by 75-years old. Moreover, there are no studies comparing the efficacy of statin intensity according to age in AMI patients treated by PCI using drug-eluting stents (DESs).

**Reference**

14.	Koopman C, Vaartjes I, Heintjes EM, Spiering W, van Dis I, Herings RM, et al. Persisting gender differences and attenuating age differences in cardiovascular drug use for prevention and treatment of coronary heart disease, 1998-2010. European heart journal. 2013;34(41):3198-205. Epub 2013/09/21. doi: 10.1093/eurheartj/eht368. PubMed PMID: 24046432.

**Reviewer #5: General Comments:
The manuscript entitled "Clinical impact of statin intensity according to age in patients with acute myocardial infarction" was reviewed. This study is a substudy from a large prospective multicenter AMI registry study in South Korea, and it shows the lack of benefit of strong statins in the elderly AMI patients. This is a very interesting study using a large volume database to compare the clinical impact of high- versus low-to-moderate-intensity statin in younger and elderly patients, has not been properly proven in AMI registry so far. In general, this manuscript is very well written. However, the reviewer would like to comment on the following two points.
Specific Comments:
1. This study used a 10-year database from 2004 to 2014, but even in the span of 10 years, there has been a further aging of the population and changes in dietary habits. In addition, the guidelines for statin administration have changed from “fire and forget” to “target to treat”, and the treatment policies of interventional cardiologists are also likely to have changed. Are there any changes in the results between the first 5 years and the second 5 years in this study?
 ANSWER** : Thank you for your comment. We additionally analyzed the result divided by 5 years (2004-2008 and 2009-2014). Interestingly, in first 5 years, there is few patients who were prescribed high-intensity statin both in younger and elderly patients. Even there was no statistical significance due to underpower, the trend was similar with our original results. However, this result would mislead our readers. If the reviewer wants, it will be reflected in the manuscript.
**Table.** Event rates and hazard ratios for clinical outcomes divided by 5 years.

| **TVF** | **Event Rates at 1-12 Month (n/%*)** | |  | |
| --- | --- | --- | --- | --- |
|  | **Low-to-moderate-intensity** | **High-intensity** | **HR (95% CI)** | **P** |
| **2004-2008** |  |  |  |  |
| **< 75 years old** | 135/1370 (10.1) | 2/27 (7.4) | 0.74 (0.18-2.99) | 0.67 |
| **≥ 75 years old** | 30/235 (12.9) | 0/5 (0) | NA | NA |
| **2009-2014** |  |  |  |  |
| **< 75 years old** | 194/3471 (5.7) | 77/1445 (5.4) | 0.96 (0.74-1.25) | 0.76 |
| **≥ 75 years old** | 101/1004 (10.3) | 38/328 (11.8) | 1.18 (0.81-1.71) | 0.39 |

**2. Based on the results of this study, is it more likely that cholesterol absorption inhibition, such as ezetimibe, will be effective in elderly patients rather than statin strength?
 ANSWER** : I appreciate your valuable comment. As we discussed this issue in the discussion session, the addition of ‘ezetimibe’, which inhibits cholesterol absorption, would be effective in elderly patients (from the results of DEBATE, IMPROVE-IT study). However, our study was designed to compare the statin-intensity. Thus, we cannot concede or refute your comment based on our study design and result.
